# Trait anxiety predicts amygdalar responses during direct processing of threat-related pictures

**DOI:** 10.1038/s41598-021-98023-7

**Published:** 2021-09-16

**Authors:** Huiyan Lin, Wolfgang H. R. Miltner, Thomas Straube

**Affiliations:** 1 Institute of Applied Psychology, School of Public Administration, Guangdong University of Finance, Guangzhou, 510521 China; 2 Institute of Medical Psychology and Systems Neuroscience, University of Muenster, 48149 Muenster, Germany; 3 Department of Clinical Psychology, Friedrich Schiller University of Jena, 07743 Jena, Germany

**Keywords:** Neuroscience, Psychology

## Abstract

Previous studies on the associations between trait anxiety and amygdalar responses to threat stimuli have resulted in mixed findings, possibly due to sample characteristics, specific tasks, and analytical methods. The present functional magnetic resonance imaging (fMRI) study aimed to investigate linear or non-linear associations between trait anxiety and amygdalar responses in a sample of participants with low, medium, and high trait anxiety scores. During scanning, participants were presented with threat-related or neutral pictures and had either to solve an emotional task or an emotional-unrelated distraction task. Results showed that only during the explicit task trait anxiety was associated with right amygdalar responses to threat-related pictures as compared to neutral pictures. The best model was a cubic model with increased amygdala responses for very low and medium trait anxiety values but decreased amygdala activation for very high trait anxiety values. The findings imply a non-linear relation between trait anxiety and amygdala activation depending on task conditions.

## Introduction

Fast and correct detection of potential threat is crucial for the survival of an organism. It has been shown repeatedly that the amygdala is a brain region, which is strongly involved in threat processing across different classes of stimuli^1–4^. It has been suggested that the amygdala classifies sensory input according to its emotional and motivational relevance^3,5^ and modulates ongoing sensory processing leading to enhanced representations of emotionally relevant stimuli^4,6,7^.

The reaction to threat varies greatly between people and depends, among other things, on the degree of trait-anxiety^8–10^. Trait anxiety is a general disposition to experience anxiety and to respond fearfully to a wide variety of unspecific threatening situations^11^. Several studies have investigated how this trait affects the amygdala activation to threat-related stimuli with mixed results. A number of studies showed that the degree of trait anxiety was positively correlated with amygdalar activation to threatening stimuli^12–22^. However, other studies reported negative correlations^23,24^ or no significant correlations^14,15,25–29^.

Discrepant findings might be due to several factors, such as sample characteristics and task conditions. With regard to sample characteristics, a large number of studies only used participants with low trait anxiety^14,15,18,30,31^ or participants with low and medium trait anxiety^12,13,17,19,21–24,26,27,29,32^. The exclusion of high trait anxiety individuals might influence the associations between trait anxiety and amygdalar activations, since there might be non-linear associations between amygdala and trait anxiety. However, such an association is only detectable when participants are investigated who span a brought range of trait anxiety from low to medium to high scores. Three studies used samples that apparently covered low, medium and high trait anxiety participants^20,25,28^. However, a closer look into the groups revealed that the sample size of the high trait anxiety group was much smaller than that of the low and medium trait anxiety groups. This dissimilar distribution of participants in the three groups might have affected the associations between trait anxiety and amygdalar activations. The associations can only be examined properly when all the three anxious groups would contain a similar number of participants per group.

In addition, task conditions have been shown to modulate relations between trait anxiety and amygdalar activation. Two studies found that trait anxiety was positively correlated with amygdalar activations to fearful faces when the experimental task was face-irrelevant as compared to a face-relevant condition^30,32^. It is notable that in these two studies, attention during the face-relevant tasks was not directed to the threatening content of the stimuli [i.e., Bishop et al.^32^: the (in)consistencies of the faces; Dickie and Armony^30^: the sex of the face]. It is unknown whether direct attention towards the threat-relevance of stimuli influences the associations between trait anxiety and amygdalar responses. At present we found no study that investigated how task conditions modulate the association between trait anxiety and amygdalar activations when using a sample of participants that spans the whole spectrum of trait anxiety.

**Figure 1:**
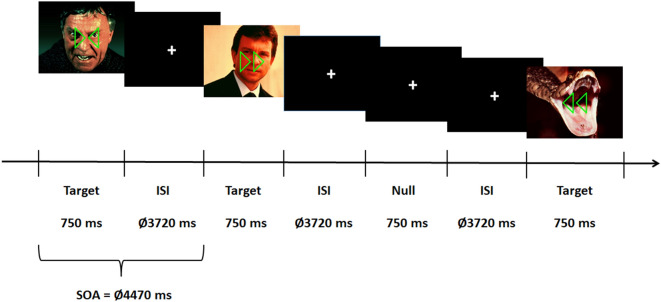
Experimental procedure. Participants were requested to identify whether the prompted picture was threat or neutral in the explicit task condition and whether the triangles that overlaid the pictures pointed to the same direction or to different directions in the implicit task condition. *SOA* stimulus onset asynchrony.

The present study aimed to investigate the associations between trait anxiety and threat-related amygdalar activation in response to aversive pictures under different task conditions. Blood oxygenation level-dependent (BOLD) activation was assessed by means of event-related functional magnetic resonance imaging (fMRI) while participants were presented either with emotionally threat-related or emotionally neutral pictures that additionally included two triangles superimposed on the pictures. The study included three similarly sized groups of individuals with either low, medium, or high trait anxiety. During the experiment, participants were asked to attend either to the emotional content of stimuli or to attend whether the included both triangles pointed simultaneously to the left or right side of the picture or pointed in opposite directions (see Fig. 1).

## Methods

### Participants

Thirty-seven healthy undergraduate students (20–25 years, *M* = 22.86, *SD* = 1.49; 19 females) were recruited from the University of Jena via public announcement in return for a compensation of 10 EUR for participation. This sample size is larger than the median sample size of previous studies investigating correlations between trait anxiety and amygdalar responses (median = 32 participants, ranging from 18 to 124)^12–32^. More importantly, participants were pre-selected according to their trait anxiety scores as described below. According to a brief interview before the start of the experiments, none of the participants reported a history of psychiatric, neurological, or other medical diseases, or reported that they had taken prescribed psychotropic medication or substance abuse that might have compromised cognitive functions. All participants were right-handed as determined by the Edinburgh Handedness Inventory^33^. All participants had normal or corrected-to-normal vision. The experimental procedure was conducted in accordance with the guidelines of ethical standards in the Declaration of Helsinki and was approved by the Ethics Committee of Faculty of Social and Behavioural Sciences of the University of Jena. Written informed consent was obtained from all participants prior to participation.

### Individual differences measures

Participants of the study were preselected from a group of 183 undergraduate students who completed the trait subscale of the State-Trait Anxiety Inventory (STAI)^11,34^ and the subscale “fear of physical harm” of the Interaktions-Angst-Fragebogen [Interaction Anxiety Questionnaire] (IAF)^35^ prior to the experiment. The trait scale of STAI reflects the degree of anxiety proneness concerning ego-threatening social situations^36–39^. It should be noted that the STAI-T is a broad negative affect measure and also seems to conflate both anxiety and depression scores^40,41^*.* The “fear of physical harm” of IAF assesses the degree of anxiety proneness when exposed to physical danger^35,36^. According to the interaction model of personality, an individual’s predisposition such as trait anxiety is related to social evaluation and physical integrity and should therefore be conceptualized as multidimensional construct^42,43^. Therefore, we created a composite measure representing trait anxiety proneness referring to both psychosocially and physically dangerous situations. The composite measure was calculated by the mean of the z-standardized raw scores of the STAI-trait scale and the scores of the IAF- “fear of physical harm” scale.

Participants were then rank-ordered according to their composite measure separately for each sex group. In each sex group, we selected 7 participants with the lowest, 7 participants with the highest, and 5 participants with the nearest to the mean score of the total group’s composite measure. The lowest and highest scores also ranked below 33% and 66%, respectively, of the total sample. This procedure resulted in a study group that fulfils the best basic requirements for the application of linear and non-linear regression models^44^.

One high-trait-anxiety male participant was excluded from statistical analysis due to excessive artefacts in the fMRI data, resulting in a sample of 18 male participants (trait anxiety score: *M* ± *SD* =  − 0.17 ± 0.79, ranging from − 1.26 to 1.24; age: *M* ± *SD* = 22.67 ± 1.33, ranging from 20 to 25 years) and 19 female participants (trait anxiety score: *M* ± *SD* = 0.16 ± 0.93, ranging from − 1.18 to 1.54; age: *M* ± *SD* = 22.05 ± 1.68, ranging from 19 to 25 years). Male and female participants did not differ in trait anxiety score or age (both *ps.* ≥ 0.228). For trait anxiety scores for each group, please refer to Table 1 for more details.

**Table 1:** Mean *z*-scores and *SD* of the composite scores of STAI-Trait and IAF for each group.

High		Medium		Low	
*M*	*SD*	*M*	*SD*	*M*	*SD*
0.92	0.45	0.08	0.22	− 0.91	0.33

### Stimuli

The stimuli included 44 coloured pictures (22 threat and 22 neutral; including 2 threat and 2 neutral for practice). Pictures were taken from the International Affective Picture System (IAPS)^45^, and adjusted to a size of 14.22 inch × 10.67 inch (horizontal × vertical) and a resolution of 72 pixel per inch. In addition, we also used average RGB-values per colour channel and compressed file sizes as a measure of luminosity and complexity, respectively^46^. Results did not show different RGB-values for all channels or compressed file sizes between threat-related and neutral pictures (*p* > 0.05). Pictures contained persons, animals, and objects (please refer to “Supplemental Materials—Table S4” for more details). Furthermore, in the foreground of the pictures, there were two triangles that were outlined in semi-transparent green. The triangles either pointed to the left or to the right of the picture frame. In one half of trials, the triangles directed to the same left or right side of the picture frame and for the other half of trials, the triangles pointed to opposing directions.

### Procedure

Presentation of stimuli and recording of behavioural responses was controlled by Presentation Software (Neruobehavioral System, Inc., Albany, CA, USA). All stimuli were shown via a back-projection screen onto an overhead mirror. All pictures and triangles were presented with dark background.

MRI-scanning was conducted in 2 runs, one in the explicit task condition and the other one in the implicit task condition. In each run, each stimulus was presented twice resulting in 320 trials (20 pictures per pictorial emotion and task × 2 pictorial emotion × 2 tasks × 2 repetitions). As shown in Fig. 1, each picture was presented for 750 ms. The presentation sequence regarding the emotion of pictures and the direction of triangles was randomized. Stimulus onset asynchrony (SOA, time between the onset of two succeeding stimuli) was 4470 ms. In the explicit task, participants were asked to indicate whether the prompted picture displayed threat or a neutral emotion. In the implicit task, participants were asked to indicate whether both triangles pointed to the same or opposite direction. This implicit task has been tested in pilot experiments and has proved to be sufficient demanding as indicated by response times. Both of the tasks emphasized response accuracy and times. Responses were given via button press of one of two buttons with the index fingers of either the left or right hand using a fiber optic response box (LUMItoucch; Photon Control). The order of tasks and the assignment of response buttons to each hand were counterbalanced across male and female participants and across participants with low, moderate, and high composite trait-anxiety scores. Additionally, 40 null stimuli (a fixation cross was presented for 750 ms and distinguished from the fixation cross seen between the presentations of the pictures) were randomly intermixed into the sequence of pictorial stimuli per task and run. No tasks were required for the null stimuli. These null stimuli resulted in temporal jittering inter-stimulus intervals^47^. Prior to each run, there were 8 practice trials to familiarize the participants with the experimental procedure. The pictures used in the practice trials were not used in the actual experiment.

After the fMRI section, participants were requested to rate the valence (1 = very unpleasant, 5 = neutral, and 9 = very pleasant), arousal (1 = very low, and 9 = very high) and threat degree (1 = not threatening at all, 9 = very threatening) of all pictures using a 9-point Likert scale to assess.

### Behavioural data recording and analysis

For behavioural data, response accuracy and times of button presses during scanning as well as ratings of emotional valence, arousal, and threat degree after scanning were recorded. Ratings on emotional valence, arousal, and threat degree were separately analysed with within-subject repeated measures analyses of variances (ANOVAs) with the factor pictorial emotion (threat versus neutral). Response accuracy and response time were analysed separately using repeated measures ANOVAs with the within-subject factors pictorial emotion (threat versus neutral) and task (explicit and implicit). We fitted regression models using the SPSS software (Version 22.0; SPSS Inc., an IBM company, Chicago, IL, USA) to investigate the linear and non-linear associations between behavioural data and trait anxiety. Note that some models require positive values for independent and/or dependent variables (e.g., logarithmic and exponential regression models). Regarding the regression models, each observed value for the related variable would add a specific constant that was larger than the absolute value of the smallest observed value, if there were non-positive observed values. A probability level of *p* < 0.05 was considered as being statistically significant. Data are expressed by mean and standard deviation (*M* ± *SD*). For analyses of response times and response accuracy, one male participant with high composite trait-anxiety scores had to be excluded due to technical problems during button presses registration.

### FMRI data acquisition and analysis

Structural and functional data were obtained using a 1.5 Tesla magnetic resonance scanner (“Magnetom Vision plus”, Siemens, Medical Systems, Erlangen, Germany) with a head coil gradient set. During the tasks, blood oxygenation level-dependent contrast functional images were acquired using a T2*-weighted echo-planar pulse sequence (TR = 2980 ms, TE = 50 ms, flip angle = 90°, field of view = 192 mm, matrix size = 64 × 64). For each participant, two scan runs of 184 volumes each during which either the indirect or direct task were realized were conducted. Each volume comprised 30 interleaved axial slices (thickness = 3 mm, gap = 1 mm, in-plane resolution = 3 × 3 mm) orientated in an approximately 30° tilted angle from the anterior–posterior commissure plane in order to reduce susceptibility artifacts in inferior parts of anterior brain areas^48^. The first four volumes of each functional run were discarded from analysis to ensure that steady-state tissue magnetization was reached. For anatomical reference, a whole brain high-resolution T1-weighted volume was recorded for each participant during the same experimental session using a 3D spoiled gradient echo pulse sequence.

Functional MRI-data preprocessing and analyses were conducted by using the software package BrainVoyager QX (Version 1.8.6; Brain Innovation, Maastricht, The Netherlands). Primarily, all volumes were realigned to the first volume in order to minimize artifacts due to head movements and a slice time correction was conducted. Further data preprocessing comprised spatial (8 mm full-width half-maximum isotropic Gaussian kernel) as well as temporal smoothing (high pass filter: 8 cycles per run, low pass filter: 2.8 s). The anatomical and functional images were co-registered and normalized to the Talairach space^49^.

Statistical analyses were performed by multiple linear regression of the signal time course at each voxel. The expected blood oxygen-level-dependent (BOLD) signal change for each event type (predictor) was modeled by a canonical hemodynamic response function. Events of interest were the two pictorial emotions (threat, neutral) and the two different task conditions (direct/indirect). Fixed-effects single participant level contrast images for planned comparisons of predictor estimates (beta weights) were entered into group-level *t* tests for a random effect analysis of the 37 participants.

Since the present study focuses on response properties of the left and right amygdalae, data analyses were conducted as regions of interest (ROIs) analysis for these brain regions. The ROIs for these brain regions were defined based on the Human-Harvard–Oxford atlas (https://scalablebrainatlas.incf.org/human/HOA06). In addition, a whole-brain analysis was performed without a priori defined ROIs. The watershed algorithm of Neuroelf (v0.9c; http://neuroelf.net/; i.e. the splitclustercoords function) was used to assess local maxima of clusters. The obtained Montreal Neurological Institute (MNI) coordinates were converted to Talairarch space using ICBM2TAL^50^.

Significant clusters were obtained through cluster-based permutation (CBP) with 1000 permutations. The non-parametric CBP method requires no assumption about the distribution of the test statistic, and results in precise false discovery rates^51^. Voxel-level threshold was set to *p* < 0.001. We investigated the effect of pictorial emotion separating for each level of task, the task-independent effect of pictorial emotion and the interaction between pictorial emotion and task. For each permutation, individual beta maps representing activation patterns in a specific effect were randomly assigned without replacement to one of the two groups. Cluster’s mass was assessed by summing all *t*-values in neighboring significant voxels. Then, the observed cluster mass was compared with the distribution of the maximum cluster mass observed in each of the 1000 permutations. Clusters masses larger than the 95% of the permutation distribution were considered as statistically significant. For the investigation of associations between trait anxiety and amygdalar activation/activations of other brain regions, the averaged differential beta values (threat-neutral) of significant clusters per task condition were fitted by linear and non-linear regression models using the SPSS software (Version 22.0; SPSS Inc., an IBM company, Chicago, IL, USA). To investigate all regression models, for each variable, observed values would be transferred into positive values by adding a specific constant, once the models require non-negative values.

## Results

### Behavioural results

#### Ratings of valence, arousal and threat

Threat-related pictures were rated as more unpleasant (*F*(1, 36) = 421.88, *p* < 0.001, ${\upeta }_{\mathrm{p}}^{2}$ = 0.92), more arousing (*F*(1, 36) = 358.48, *p* < 0.001, ${\upeta }_{\mathrm{p}}^{2}$ = 0.91), and more threatening (*F*(1, 36) = 897.52, *p* < 0.001, ${\upeta }_{\mathrm{p}}^{2}$ = 0.96) than neutral pictures. See descriptive data in Table 2.

**Table 2:** Mean ratings of valence, arousal, and threat degree for each picture type and the *SD*.

	Threat		Neutral	
	*M*	*SD*	*M*	*SD*
Valence	2.35	0.71	5.66	0.88
Arousal	6.59	1.26	2.38	0.89
Threat	7.12	1.10	1.87	0.67

#### Response accuracy and times

##### Effects of task and pictorial emotion on response accuracy and times

The results of response accuracy only showed a main effect of factor task (*F*(1, 35) = 19.19, *p* < 0.001, ${\upeta }_{\mathrm{p}}^{2}$ = 0.35). Response accuracy was generally better in the implicit condition than in the explicit condition. Other main effect or interaction was not significant (both *ps.* ≥ 0.429). With regard to response times, the ANOVA did not show any main effects or interaction (all *ps.* ≥ 0.136). See descriptive data in Table 3.

**Table 3:** Mean response accuracy (%) and time (ms) and *SD* for each experimental condition.

	Threat		Neutral	
	*M*	*SD*	*M*	*SD*
**Response accuracy**				
Explicit	91.88	9.66	92.08	5.36
Implicit	95.21	4.49	96.53	4.60
**Response times**				
Explicit	706.80	140.10	706.18	116.51
Implicit	689.83	105.04	674.76	94.67

##### The relationships between trait anxiety and response accuracy/times

**Figure 2:**
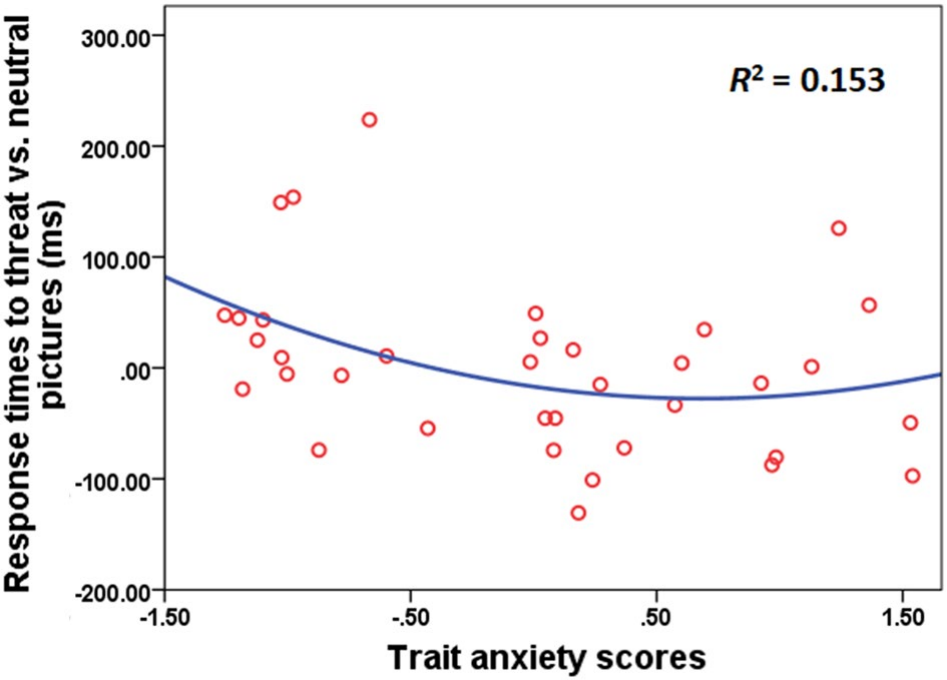
The relation between trait anxiety and differential responses times between threat and neutral pictures in the explicit condition. The fittest model was the quadratic model.

In the explicit condition, regression analysis showed linear and non-linear associations between trait anxiety and response times, with the highest fit for the quadratic association (Linear: y =  − 29.07x + 189.58, *F*(1, 34) = 4.25, *p* = 0.047, *R*^2^ = 0.111; Logarithmic: y =  − 56.13ln(x) + 164.24, *F*(1, 34) = 5.09, *p* = 0.031, *R*^2^ = 0.130; Inverse: y = 85.63/x + 77.55, *F*(1, 34) = 5.20, *p* = 0.029, *R*^2^ = 0.133; Quadratic: y = 23.06x^2^ − 123.77x + 269.42, *F*(2, 33) = 2.98, *p* = 0.065, *R*^2^ = 0.153; Fig. 2). There were no models found to be significant between trait anxiety and response accuracy in the explicit condition and between trait anxiety and response accuracy/times in the implicit condition (*p* > 0.05).

### FMRI results

#### ROI analysis

For both, left and right amygdala, there was a task-independent effect of pictorial emotion, with higher activation for threat than for neutral pictures (left amygdala: peak voxel coordinates: x =  − 23, y =  − 4, z =  − 13; size: 2427 voxels; maximal *t*-value = 7.48; averaged *t*-value = 5.17; *p* < 0.05 corrected; right amygdala: x = 25, y =  − 3, z =  − 12; size: 1136 voxels; maximal *t*-value = 5.53; averaged *t*-value = 4.15; *p* < 0.05 corrected). Moreover, the effect of pictorial emotion was also significant in both the explicit condition (left amygdala: peak voxel coordinates: x =  − 23, y =  − 5, z =  − 12; size: 1768 voxels; maximal *t*-value = 6.36; averaged *t*-value = 4.60; *p* < 0.05 corrected; right amygdala: x = 20, y =  − 6, z =  − 7; size: 71 voxels; maximal *t*-value = 4.65; averaged *t*-value = 3.75; *p* < 0.05 corrected) and the implicit condition (left amygdala: peak voxel coordinates: x =  − 25, y =  − 3, z =  − 13; size: 1206 voxels; maximal *t*-value = 5.16; averaged *t*-value = 3.80; *p* < 0.05 corrected; right amygdala: x = 26, y = − 2, z = -− 15; size: 812 voxels; maximal *t*-value = 5.13; averaged *t*-value = 4.17; *p* < 0.05 corrected). There was no significant interaction between emotion and task.

#### Whole brain analysis

There were a great number of brain regions showing task-dependent and task-independent emotional effects. Specific activation brain regions are found in “Supplemental Materials—Tables S5–S8”.

#### The relationships between trait anxiety and brain activity

##### The relationships between trait anxiety and amygdalar responses

**Figure 3:**
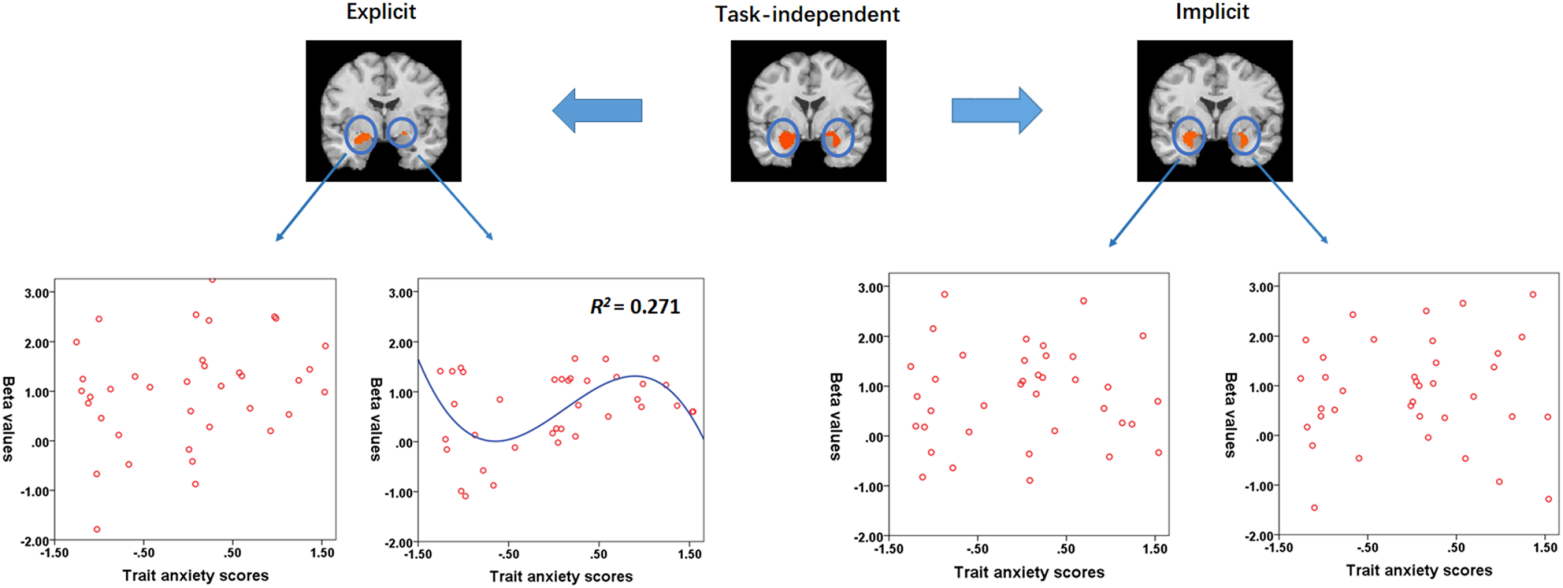
The upper panel: the task-dependent and independent effects of pictorial emotion on amygdalar responses. The lower panel: the relation between trait anxiety and amygdalar responses to threat vs. neutral pictures. A cubic model was the best fit for the right amygdala in the explicit condition. There were no significant models for the left amygdala or the implicit condition. Results only showed an association between trait anxiety and right amygdalar responses in the explicit condition.

In the explicit condition, regression analyses showed a significant association between trait anxiety scores and right amygdalar responses to threat-related as compared to neutral pictures in several models (i.e., cubic, linear, compound, growth and exponential), whereas *R*^2^ was largest for the cubic model (cubic: y =  − 0.71x^3^ + 3.18x^2^ − 3.45x + 2.30, *F*(3, 33) = 4.09, *p* = 0.014, *R*^2^ = 0.271; linear: y = 0.30x + 1.43, *F*(1, 35) = 4.55, *p* = 0.040, *R*^2^ = 0.115; compound: y = 0.99 × 1.39^x^, *F*(1, 35) = 6.10, *p* = 0.019, *R*^2^ = 0.148; growth: y = *e*^−0.01+0.330x^, *F*(1, 35) = 6.10, *p* = 0.019, *R*^2^ = 0.148; exponential: y = 0.99 × *e*^0.330x^, *F*(1, 35) = 6.10, *p* = 0.019, *R*^2^ = 0.148). See the cubic model in Fig. 3 and other models in “Supplemental Materials—Fig. S3”. Moreover, this association was not specific to the measures of trait anxiety, as the cubic relation was also the fittest model even when the association was analysed by the scores of either STAI-T [minimum *R*^2^ < 0.01, maximum *R*^2^ (cubic): 0.138] or IAF [minimum *R*^2^ = 0.01, maximum *R*^*2*^ (cubic): 0.186]. Additionally, in order to understand whether the models were related to threat-related pictures, neutral pictures or the differences between these pictures, we also examined the correlations between trait anxiety and amygdalar responses to threat-related/neutral pictures vs. baseline, and the regression analysis did not show any significant models (*p* > 0.05). Thus, trait anxiety was associated with amygdalar responses to the differences between threat-related and neutral pictures rather than to either of the pictures. Other model fittings (logarithmic, inverse, quadratic, power and S) were not significant (*p* > 0.05). Regression analyses did not show significant results for the amygdala during the implicit condition (*p* > 0.05).

##### The relationships between trait anxiety and activity of other brain regions

Also for other areas, significant linear and non-linear relations were found specifically during both explicit and implicit conditions and the non-linear associations showed consistently the best fit in the explicit condition. Please refer to the specific model for each brain region in “Supplemental Materials—Tables S6 and S7”.

## Discussion

The present study investigated linear and nonlinear associations between trait anxiety and threat-related amygdalar responses under different attentional conditions. We found that only the explicit emotional condition revealed an association between trait anxiety and amygdala activation. The best regression model indicated a cubic relation with low and medium values of trait anxiety leading to highest amygdala activations, irrespective of whether trait anxiety was measured by the scores of STAI-T or IAF or their composite scores. These findings imply that trait anxiety influences threat-related amygdalar responses in a non-linear way, especially when the threat-relevance of stimuli is attended.

The amygdala is thought to be a crucial brain region in coding threat across sensory modalities^1–4^. Moreover, previous studies have suggested that this threat-related amygdalar activation takes place irrespective of whether attention is shifted to the emotional or non-emotional content of stimuli^5,52–55^. In line with previous studies, the present study also found increased amygdalar responses to threat-related pictures when attention was directed to both the threat-relevance and threat-irrelevance of the stimuli.

More importantly, the present study found that the threat-related amygdalar responses were predicted by the degree of trait anxiety. However, the findings revealed a complex non-linear pattern and this pattern was limited to the explicit condition. As outlined in the “Introduction” section, there are no consistent findings regarding the associations between trait anxiety and amygdalar activations^12–29^. This might be due to a true (absent or variable) relationship between trait anxiety and amygdalar activations (see the following paragraph for details), or to specific characteristics of samples, stimuli, and task conditions. Moreover, previous studies did not investigate whether direct attention towards the threat-relevance of stimuli influences the results. In the current study, we attempted to include the whole spectrum of trait anxiety. We also varied attentional conditions by requiring participants performing an emotion-related task or an emotion-unrelated task.

Several studies did not show a consistent finding, even though sample characteristics and experimental tasks were similar^12,20,21,25^. Previous studies only investigated the linear association between trait anxiety and threat-related amygdalar responses^12–29^. The linear association might not reflect the true relation between trait anxiety and amygdalar activations. During the explicit condition of the present study, we also observed a significant linear regression, but this model showed not the best fit compared to non-linear models.

The best fit was observed for the cubic model, which was not specific to the measures of trait anxiety (STAI-T, IAF or the composite). While this relation has to be confirmed in future studies, a cubic association might reflect different adaptive mechanisms depending on trait anxiety. It has been demonstrated that high trait anxious individuals have a stronger anxious experience and a higher vigilance and apprehension in perceiving threatening and uncertain situations and thus have a processing bias for threatening environmental cues^56,57^. This processing bias might be accompanied with an increased amygdalar responses, particularly when the degree of trait anxiety is not extremely high or low. However, extremely high trait anxiety might result in excessive vigilance and uncontrollable sensitivity to threatening situations and increased susceptibility for forming affective disorders^58–60^. Thus, for high trait anxiety individuals, reducing vigilance and sensitivity to threatening stimuli to some extents might be helpful in reducing risks in developing affective disorders. The decreased excessive vigilance and sensitivity might lead to the reduction of amygdalar responses. Furthermore, high trait anxiety has been associated with reduced discrimination of threatening and safety stimuli^61^, which would explain reduced differential amygdala activations to threat vs neutral pictures. With regard to individuals with low trait anxiety, increased amygdalar activations might be associated with differential processing of pictorial threat and neutral stimuli, but these individuals might be nevertheless insensitive to certain potentially threatening situations^62^, possibly due to altered brain activations outside the amygdala.

Notably, as is shown in Fig. 2, the explicit condition revealed high variance of right amygdalar values for low and medium trait anxiety but low variance for high trait anxiety. This pattern of variance might generally lead to higher amygdalar activation for high trait anxiety individuals. Additionally, the regression analyses revealed a significant fit not only for the best fitting cubic model but for several other models (linear, compound, growth, and exponential). Thus, we cannot exclude the possibility of unspecific relationships between trait anxiety and amygdalar responses or a better fit for other models with increased sample sizes. Future studies might further investigate this related issue.

Moreover, the significant association between trait anxiety and threat-related amygdalar response was found only when attention was shifted to the emotional content of stimuli. Trait anxiety reflects the individual disposition to experience fear/anxiety-relevant feelings or thoughts and to show anxiety-related behaviours^11^. In the present study, directing to the threatening portions of the stimuli in the explicit condition might allow participants to have a stronger fearful experience and to produce more potentially withdrawal behaviours. This might result in a stronger association between trait anxiety degree and threat-related amygdalar responses, at least in response to complex emotional scenes. Finally, this finding was only observed for the right amygdala. This might represent a threshold effect or underline a specific role of the right amygdala for the understanding of trait anxiety.

The current findings were partially in line with two previous studies^30,32^, which indicated that task conditions influenced the associations between trait anxiety and amygdalar responses. However, different from the current findings, Bishop et al.^32^ reported that shifting attention away from threat-related stimuli led to a stronger association. Dickie and Armony^30^ showed reversed associations when attention is shifted to or away from the threat-related stimuli. In these two studies, only the non-emotional content was processed even though attention was directed to target stimuli. However, the explicit condition in the present study required direct processing of the threatening content of target stimuli, which might affect the results of the studies. Furthermore, stimulus complexity was different across studies (emotional faces in Bishop et al.’s study^32^, scene-emotional face composites in Dickie and Armony’s study^30^ and complex emotional pictures in the current study), which might lead to different attentional requirements to see associations between trait anxiety and amygdalar activations.

In addition, the present study also investigated the associations between trait anxiety and behavioural data as well as between trait anxiety and activations of other brain regions for threat vs. neutral pictures. In fact, previous studies have found linear associations between trait anxiety and behavioural data^14,15,30,63^ and between trait anxiety and activations of several other brain regions (e.g., prefrontal cortex, anterior cingulate cortex, insula, precuneus, middle temporal gyrus, thalamus)^19,20,25,64–66^, whereas it is still unclear whether these associations are affected by task demands and/or whether the associations are fitted better by linear or non-linear models. For the present study, we observed that the associations between trait anxiety and response times for threat vs. neutral pictures were likely to be non-linear in the explicit condition. For neural activity, the results revealed linear and non-linear associations between trait anxiety and activations of several other brain regions for threat vs. neutral pictures in both explicit and implicit conditions, and the non-linear associations always showed the best fit in the explicit condition. Therefore, the findings in the present study might give new insights that the associations between trait anxiety and behavioural data/brain activity are dependent on task demands and are not always reflected by the simple linear model but rather by complex non-linear models.

Finally, there are some limitations of the present study that suggest outlines for future studies. Even though we tried to cover the whole trait anxiety range and sample size was larger than in most previous studies, future studies might increase sample size and sampling of trait anxiety scores. Furthermore, it has been proposed that connectivity measures might be more strongly related to trait anxiety than amygdalar activations^25,26,67^. However, this analysis was beyond the scope of the current manuscript and data acquisition. In the present study, BOLD responses in amygdala were recorded during the presentation of emotional pictures. It is unclear whether a similar association would be shown when some other visual stimuli (e.g., faces and words) or stimuli from other sensory modalities (e.g., voices) are used. Fourth, recent studies suggested that the STAI-Trait scale might reflect not only the degree of anxiety but also that of a broad negative affect (e.g., depression)^8,68^. Thus, in the present study, the associations between trait anxiety and amygdalar responses fitting in several models might be affected by this factor, even though we used a more suited composite score of different trait anxiety measures. Future studies might use the State-Trait Inventory for Cognitive and Somatic Anxiety^40,41^ that is more specific to anxiety to further investigate the related issue. Finally, in addition to task demands, previous studies reported that the associations between trait anxiety and amygdalar responses were modulated by several other factors, e.g., social support^16^ and attachment-security^27^. Future studies might include these factors and further investigate the associations between trait anxiety and amygdalar responses to threat.

## Conclusion

The present study provides evidence that direct attention to threatening pictures leads to increased amygdalar activations for very low and medium trait anxiety individuals but decreased for very high trait anxiety individuals. The finding suggests a non-linear relation between trait anxiety and threat-related amygdalar activations depending on task relevance during the processing of emotional pictures.

## Supplementary Information

Supplementary Information.
