# Supplementary material for: Trait anxiety predicts amygdalar responses during direct processing of threat-related pictures
Source: Sci Rep. 2021 Sep 16;11:18469. doi: 10.1038/s41598-021-98023-7 (PMC8446049; doi:10.1038/s41598-021-98023-7)
Supplement: Supplementary file 1 — Supplementary Information. [file 41598_2021_98023_MOESM1_ESM.docx]

**Supplemental materials**

**Table 4.** The number and descriptions of IAPS.

| Threatening pictures | | Neutral pictures | |
| --- | --- | --- | --- |
| IAPS No. | Description | IAPS No. | Description |
| 1120 | Snake | 1616 | Bird |
| 1300 | Pit Bull | 1670 | Cow |
| 1930 | Shark | 1945 | Turtle |
| 2120 | Angry Face | 2190 | Man |
| 3010 | Mutilation | 2200 | Neutral Face |
| 3071 | Mutilation | 2214 | Neutral Man |
| 3100 | Burn Victim | 2221 | Judge |
| 3120 | Dead Body | 2372 | Woman |
| 3130 | Mutilation | 2383 | Secretary |
| 3530 | Attack | 2410 | Boy |
| 3550.1 | Plane Crash | 2575 | Propeller |
| 6230 | Aimed Gun | 2749 | Smoking |
| 6250.1 | Aimed Gun | 2830 | Woman |
| 6260 | Aimed Gun | 2840 | Chess |
| 6300 | Knife | 2850 | Tourist |
| 6313 | Attack | 7010 | Basket |
| 6350 | Attack | 7175 | Lamp |
| 6370 | Attack | 7503 | Card Dealer |
| 6550 | Attack | 7550 | Office |
| 9410 | Soldier | 8010 | Runner |

**Table 5.** Significant activations for task-independent emotional effects

| Regions | Laterality | Talairach coordinates of peak voxel | | | k | *t* value maximum | *t* value average |
| --- | --- | --- | --- | --- | --- | --- | --- |
|  |  | x | y | z |  |  |  |
| **Cluster1** | L/R | -38 | -67 | -9 | 36858 | 11.46 | 5.06 |
| Inferior Frontal Gyrus | L | -49 | 25 | 21 | 374 | 9.55 | 6.43 |
| Inferior Frontal Gyrus | L | -34 | 30 | 4 | 373 | 6.38 | 4.69 |
| Inferior Frontal Gyrus | L | -46 | 19 | 11 | 318 | 7.82 | 5.69 |
| Middle Frontal Gyrus | L | -48 | 16 | 26 | 404 | 9.01 | 6.05 |
| Middle Frontal Gyrus | L | -41 | 5 | 41 | 324 | 7.26 | 4.84 |
| Middle Frontal Gyrus | L | -29 | 40 | 21 | 309 | 6.97 | 4.88 |
| Middle Frontal Gyrus | L | -34 | -9 | 46 | 167 | 6.80 | 4.65 |
| Middle Frontal Gyrus | L | -26 | 39 | 40 | 284 | 7.75 | 4.89 |
| Medial Frontal Gyrus | L | -4 | 46 | 28 | 251 | 8.41 | 6.16 |
| Medial Frontal Gyrus | L | -8 | 11 | 43 | 196 | 6.30 | 4.73 |
| Medial Frontal Gyrus | L | -17 | -13 | 46 | 99 | 5.57 | 4.30 |
| Superior Frontal Gyrus | L | -38 | 47 | 21 | 402 | 7.82 | 5.10 |
| Superior Frontal Gyrus | L | -19 | 54 | 27 | 342 | 7.89 | 5.24 |
| Superior Frontal Gyrus | L | -4 | 7 | 53 | 300 | 7.15 | 4.81 |
| Superior Frontal Gyrus | L | 0 | 54 | 27 | 230 | 7.58 | 5.42 |
| Cingulate Gyrus | L | -15 | 26 | 25 | 338 | 7.83 | 4.82 |
| Cingulate Gyrus | L | -4 | 35 | 29 | 213 | 7.96 | 6.50 |
| Cingulate Gyrus | L | -4 | 23 | 35 | 233 | 7.91 | 5.68 |
| Cingulate Gyrus | L | -3 | -51 | 28 | 201 | 4.91 | 4.06 |
| Cingulate Gyrus | L | -10 | -15 | 31 | 180 | 6.69 | 5.09 |
| Cingulate Gyrus | L | -17 | -1 | 40 | 178 | 6.02 | 4.53 |
| Cingulate Gyrus | L | -14 | -1 | 51 | 167 | 5.28 | 4.13 |
| Cingulate Gyrus | L | -22 | -34 | 37 | 158 | 6.20 | 4.66 |
| Cingulate Gyrus | L | -4 | 11 | 32 | 140 | 6.60 | 5.02 |
| Cingulate Gyrus | L | -16 | -24 | 40 | 127 | 6.58 | 4.74 |
| Cingulate Gyrus | L | -6 | -46 | 44 | 48 | 4.34 | 3.78 |
| Posterior Cingulate | L | -6 | -39 | 18 | 276 | 6.32 | 4.37 |
| Insula | L | -45 | -13 | 20 | 407 | 6.48 | 4.53 |
| Insula | L | -35 | 3 | 9 | 288 | 6.98 | 5.21 |
| Insula | L | -28 | -25 | 15 | 206 | 6.11 | 4.40 |
| Insula | L | -45 | -15 | 1 | 182 | 5.69 | 4.56 |
| Insula | L | -31 | -9 | 20 | 147 | 5.78 | 4.85 |
| Claustrum | L | -29 | 12 | -2 | 581 | 9.18 | 5.17 |
| Claustrum | L | -37 | -8 | -4 | 228 | 5.81 | 4.62 |
| Claustrum | L | -28 | -17 | 20 | 130 | 5.57 | 4.39 |
| Precentral Gyrus | L | -46 | 1 | 26 | 287 | 7.39 | 5.50 |
| Precentral Gyrus | L | -35 | -3 | 31 | 225 | 7.28 | 5.24 |
| Precentral Gyrus | L | -42 | -7 | 41 | 165 | 6.93 | 4.90 |
| Precentral Gyrus | L | -25 | -16 | 44 | 147 | 6.77 | 4.98 |
| Precentral Gyrus | L | -57 | 5 | 5 | 58 | 4.27 | 3.58 |
| Postcentral Gyrus | L | -44 | -24 | 43 | 244 | 5.19 | 4.09 |
| Inferior Parietal Lobule | L | -31 | -32 | 38 | 147 | 6.29 | 4.74 |
| Superior Parietal Lobule | L | -30 | -52 | 45 | 192 | 5.92 | 4.49 |
| Precuneus | L | -22 | -71 | 24 | 371 | 7.39 | 5.25 |
| Precuneus | L | -22 | -62 | 37 | 226 | 6.46 | 4.90 |
| Precuneus | L | -7 | -69 | 21 | 223 | 6.32 | 4.87 |
| Precuneus | L | -30 | -64 | 41 | 213 | 6.49 | 4.60 |
| Precuneus | L | -3 | -51 | 36 | 80 | 4.48 | 3.78 |
| Supramarginal Gyrus | L | -42 | -41 | 37 | 301 | 6.64 | 4.69 |
| Middle Temporal Gyrus | L | -50 | -56 | 10 | 485 | 8.62 | 5.73 |
| Middle Temporal Gyrus | L | -40 | -56 | 10 | 402 | 8.56 | 6.10 |
| Superior Temporal Gyrus | L | -50 | -45 | 21 | 589 | 7.76 | 4.98 |
| Superior Temporal Gyrus | L | -53 | -27 | 3 | 241 | 6.30 | 4.22 |
| Superior Temporal Gyrus | L | -40 | -32 | 2 | 127 | 4.40 | 3.82 |
| Superior Temporal Gyrus | L | -54 | -3 | -3 | 103 | 5.22 | 4.05 |
| Superior Temporal Gyrus | L | -55 | 5 | -3 | 69 | 4.26 | 3.75 |
| Fusiform Gyrus | L | -38 | -67 | -9 | 870 | 11.46 | 6.97 |
| Fusiform Gyrus | L | -40 | -49 | -8 | 703 | 9.94 | 6.36 |
| Cuneus | L | -22 | -84 | 12 | 363 | 7.41 | 5.64 |
| Cuneus | L | -10 | -79 | 25 | 258 | 7.38 | 5.19 |
| Cuneus | L | -13 | -77 | 14 | 234 | 7.12 | 5.66 |
| Cuneus | L | -16 | -86 | 22 | 112 | 6.39 | 5.04 |
| Lingual Gyrus | L | -13 | -86 | 2 | 476 | 7.14 | 4.74 |
| Lingual Gyrus | L | -19 | -55 | 2 | 442 | 7.14 | 4.96 |
| Lentiform Nucleus | L | -28 | -19 | 5 | 149 | 5.46 | 4.26 |
| Parahippocampal Gyrus | L | -24 | -22 | -9 | 63 | 4.33 | 3.75 |
| Caudate | L | -16 | -2 | 15 | 373 | 7.31 | 4.80 |
| Thalamus | L | -18 | -32 | 5 | 172 | 6.20 | 4.64 |
| Thalamus | L | -3 | -15 | 19 | 170 | 5.10 | 4.11 |
| Thalamus | L | -15 | -18 | 12 | 147 | 6.56 | 4.93 |
| Thalamus | L | -12 | -21 | 4 | 145 | 5.50 | 4.32 |
| Thalamus | L | -3 | -28 | 5 | 123 | 4.81 | 3.93 |
| Anterior Lobe | L | -2 | -43 | -25 | 52 | 3.86 | 3.53 |
| Anterior Lobe | L | -16 | -43 | -26 | 41 | 3.94 | 3.49 |
| Culmen | L | 0 | -54 | 0 | 333 | 6.14 | 4.46 |
| Culmen | L | -25 | -30 | -21 | 245 | 5.66 | 4.27 |
| Culmen | L | -5 | -27 | -15 | 44 | 4.32 | 3.75 |
| Culmen | L | -5 | -34 | -19 | 26 | 3.71 | 3.46 |
| Culmen | L | -34 | -39 | -30 | 15 | 3.70 | 3.50 |
| Sub-Gyral | L | -34 | -41 | 35 | 210 | 6.01 | 4.73 |
| Sub-Gyral | L | -33 | -22 | -9 | 78 | 4.91 | 4.01 |
| Inferior Frontal Gyrus | R | 54 | 17 | 19 | 380 | 9.04 | 5.77 |
| Inferior Frontal Gyrus | R | 36 | 7 | 31 | 267 | 7.54 | 5.63 |
| Inferior Frontal Gyrus | R | 40 | 25 | 8 | 237 | 7.06 | 5.36 |
| Inferior Frontal Gyrus | R | 33 | 16 | -16 | 66 | 5.40 | 3.99 |
| Middle Frontal Gyrus | R | 49 | 27 | 23 | 385 | 8.82 | 5.87 |
| Middle Frontal Gyrus | R | 29 | 30 | 30 | 256 | 5.85 | 4.54 |
| Middle Frontal Gyrus | R | 36 | 44 | 21 | 241 | 7.26 | 5.34 |
| Middle Frontal Gyrus | R | 38 | 38 | 6 | 194 | 6.93 | 4.79 |
| Medial Frontal Gyrus | R | 11 | 1 | 53 | 243 | 6.10 | 4.36 |
| Superior Frontal Gyrus | R | 8 | 42 | 42 | 383 | 6.35 | 4.56 |
| Superior Frontal Gyrus | R | 26 | 51 | 25 | 273 | 8.12 | 5.37 |
| Superior Frontal Gyrus | R | 16 | 56 | 27 | 213 | 7.85 | 5.31 |
| Cingulate Gyrus | R | 3 | -21 | 31 | 241 | 6.72 | 4.93 |
| Cingulate Gyrus | R | 7 | 5 | 40 | 227 | 6.25 | 4.72 |
| Cingulate Gyrus | R | 7 | 20 | 27 | 193 | 7.80 | 5.87 |
| Cingulate Gyrus | R | 21 | 12 | 32 | 178 | 7.73 | 4.85 |
| Cingulate Gyrus | R | 16 | -38 | 31 | 157 | 5.27 | 4.09 |
| Cingulate Gyrus | R | 7 | 10 | 26 | 114 | 7.44 | 5.48 |
| Anterior Cingulate | R | 22 | 37 | 10 | 449 | 6.83 | 4.71 |
| Posterior Cingulate | R | 22 | -61 | 11 | 187 | 6.71 | 5.08 |
| Posterior Cingulate | R | 9 | -34 | 23 | 168 | 5.94 | 4.38 |
| Insula | R | 31 | -34 | 21 | 233 | 7.22 | 4.66 |
| Insula | R | 42 | -23 | 1 | 225 | 5.85 | 4.33 |
| Insula | R | 30 | 14 | 16 | 197 | 6.51 | 4.99 |
| Insula | R | 32 | -2 | 21 | 193 | 7.49 | 5.61 |
| Insula | R | 37 | -17 | 13 | 184 | 4.99 | 4.13 |
| Claustrum | R | 35 | 7 | 4 | 217 | 7.74 | 5.05 |
| Claustrum | R | 34 | -4 | -8 | 198 | 5.53 | 4.23 |
| Precentral Gyrus | R | 46 | -4 | 31 | 329 | 6.72 | 4.92 |
| Precentral Gyrus | R | 46 | -9 | 41 | 320 | 6.75 | 4.38 |
| Precentral Gyrus | R | 50 | 6 | 7 | 201 | 5.97 | 4.56 |
| Precentral Gyrus | R | 29 | -7 | 30 | 140 | 4.66 | 3.96 |
| Postcentral Gyrus | R | 53 | -20 | 27 | 217 | 5.56 | 4.33 |
| Postcentral Gyrus | R | 28 | -21 | 35 | 191 | 6.10 | 4.17 |
| Postcentral Gyrus | R | 62 | -20 | 24 | 157 | 5.57 | 4.07 |
| Inferior Parietal Lobule | R | 45 | -36 | 31 | 208 | 6.17 | 4.27 |
| Inferior Parietal Lobule | R | 61 | -42 | 25 | 74 | 4.27 | 3.74 |
| Precuneus | R | 28 | -73 | 27 | 357 | 6.30 | 4.70 |
| Precuneus | R | 25 | -62 | 23 | 251 | 7.15 | 5.11 |
| Angular Gyrus | R | 34 | -58 | 38 | 185 | 5.98 | 4.32 |
| Middle Temporal Gyrus | R | 37 | -53 | 23 | 146 | 5.93 | 4.64 |
| Middle Temporal Gyrus | R | 59 | -27 | 1 | 78 | 4.54 | 3.81 |
| Superior Temporal Gyrus | R | 48 | -38 | 7 | 309 | 7.31 | 5.10 |
| Superior Temporal Gyrus | R | 50 | -45 | 16 | 256 | 6.53 | 4.68 |
| Superior Temporal Gyrus | R | 37 | -53 | 14 | 203 | 6.18 | 4.93 |
| Superior Temporal Gyrus | R | 48 | -15 | 2 | 146 | 5.45 | 4.07 |
| Superior Temporal Gyrus | R | 41 | 10 | -15 | 139 | 5.08 | 4.15 |
| Superior Temporal Gyrus | R | 48 | -17 | -6 | 133 | 5.81 | 4.14 |
| Superior Temporal Gyrus | R | 53 | 9 | -3 | 87 | 4.75 | 3.90 |
| Fusiform Gyrus | R | 37 | -53 | -9 | 549 | 10.89 | 7.19 |
| Fusiform Gyrus | R | 45 | -63 | -6 | 345 | 10.58 | 6.96 |
| Inferior Occipital Gyrus | R | 40 | -72 | -1 | 347 | 10.33 | 6.73 |
| Middle Occipital Gyrus | R | 42 | -70 | 11 | 429 | 8.61 | 6.08 |
| Cuneus | R | 11 | -88 | 10 | 381 | 8.23 | 5.73 |
| Cuneus | R | 9 | -73 | 16 | 350 | 7.77 | 5.68 |
| Lingual Gyrus | R | 21 | -83 | 6 | 400 | 6.81 | 5.02 |
| Lingual Gyrus | R | 22 | -56 | 2 | 262 | 6.89 | 5.48 |
| Lentiform Nucleus | R | 28 | -10 | 4 | 163 | 6.33 | 4.91 |
| Lentiform Nucleus | R | 16 | 2 | 8 | 162 | 6.30 | 4.38 |
| Lentiform Nucleus | R | 22 | -7 | 11 | 107 | 6.51 | 5.17 |
| Parahippocampal Gyrus | R | 18 | -37 | 4 | 225 | 6.50 | 4.62 |
| Caudate | R | 16 | 0 | 19 | 145 | 5.39 | 4.27 |
| Thalamus | R | 6 | -14 | -1 | 217 | 5.52 | 4.22 |
| Thalamus | R | 21 | -23 | 8 | 183 | 6.59 | 5.01 |
| Thalamus | R | 19 | -14 | 15 | 137 | 6.54 | 5.10 |
| Thalamus | R | 9 | -18 | 14 | 97 | 4.66 | 3.91 |
| Midbrain Red Nucleus | R | 6 | -17 | -10 | 54 | 4.77 | 3.95 |
| Anterior Lobe | R | 33 | -51 | -29 | 117 | 6.29 | 4.94 |
| Anterior Lobe | R | 6 | -40 | -26 | 13 | 3.79 | 3.44 |
| Culmen | R | 0 | -58 | -18 | 467 | 5.88 | 4.16 |
| Culmen | R | 21 | -56 | -19 | 287 | 7.97 | 5.66 |
| Culmen | R | 8 | -40 | -9 | 275 | 6.50 | 4.69 |
| Culmen | R | 42 | -43 | -28 | 190 | 6.00 | 4.51 |
| Culmen | R | 6 | -27 | -15 | 37 | 3.88 | 3.51 |
| Tuber | R | 42 | -65 | -27 | 147 | 6.90 | 5.14 |
| Uvula | R | 28 | -66 | -25 | 226 | 7.46 | 5.57 |
| Pyramis | R | 12 | -69 | -23 | 228 | 7.49 | 4.74 |
| Sub-Gyral | R | 31 | -47 | 31 | 141 | 6.14 | 4.58 |
| **Cluster2**-Anterior Cingulate | R | 3 | 22 | -2 | 27 | 4.51 | 3.78 |

**Table 6.** Significant activations for emotional contrasts in the explicit condition and the associations with trait anxiety

| **Significant activations for emotional contrasts in the explicit condition** | | | | | | | | **Associations with trait anxiety** | | |
| --- | --- | --- | --- | --- | --- | --- | --- | --- | --- | --- |
| Regions | Laterality | Talairach coordinate of peak voxel | | | k | *t* value maximum | *t* value average | *R*^2^ | Statistical values | Model(s) |
|  |  | x | y | z |  |  |  |  |  |  |
| **Cluster1** | L/R | 39 | -45 | -5 | 18123 | 8.05 | 4.23 |  |  |  |
| Inferior Frontal Gyrus | L | -43 | 23 | 11 | 223 | 6.36 | 4.51 |  |  |  |
| Inferior Frontal Gyrus | L | -35 | 36 | 14 | 136 | 4.74 | 3.99 |  |  |  |
| Inferior Frontal Gyrus | L | -41 | 4 | 24 | 124 | 5.45 | 4.25 |  |  |  |
| Inferior Frontal Gyrus | L | -31 | 33 | 6 | 44 | 4.05 | 3.67 |  |  |  |
| Inferior Frontal Gyrus | L | -23 | 15 | -13 | 13 | 3.52 | 3.35 | 0.197 | *F*(1, 34) = 4.17, *p* = 0.024 | Quadratic: y = -0.32x^2^ + 1.33x + 3.10 |
|  |  |  |  |  |  |  |  | 0.149 | *F*(1, 35) = 6.13, *p* = 0.018 | Linear: y = -0.44x + 3.50 |
|  |  |  |  |  |  |  |  | 0.157 | *F*(1, 35) = 6.51, *p* = 0.015 | Logarithmic: y = 0.41ln(x) + 4.10 |
|  |  |  |  |  |  |  |  | 0.146 | *F*(1, 35) = 5.99, *p* = 0.020 | Compound: y = 3.35 × 1.13^x^ |
|  |  |  |  |  |  |  |  |  |  | Growth: y = *e*^0.12x + 1.21^ |
|  |  |  |  |  |  |  |  |  |  | Exponential: y = *e*^0.12x^ × 3.35 |
| Middle Frontal Gyrus | L | -49 | 34 | 22 | 261 | 6.21 | 4.75 |  |  |  |
| Middle Frontal Gyrus | L | -50 | 15 | 26 | 200 | 6.35 | 4.74 |  |  |  |
| Middle Frontal Gyrus | L | -46 | 25 | 26 | 140 | 6.70 | 5.17 |  |  |  |
| Middle Frontal Gyrus | L | -23 | 37 | 37 | 137 | 5.49 | 4.01 |  |  |  |
| Middle Frontal Gyrus | L | -41 | 5 | 41 | 89 | 5.56 | 4.45 |  |  |  |
| Middle Frontal Gyrus | L | -30 | 3 | 36 | 87 | 4.93 | 4.03 | 0.235 | *F*(1, 35) = 10.74, *p* = 0.002 | S: y = *e*^(-0.07/x + 1.49)^ |
|  |  |  |  |  |  |  |  | 0.164 | *F*(1, 35) = 6.86, *p* = 0.013 | Inverse: y = -0.19/x + 4.40 |
|  |  |  |  |  |  |  |  | 0.110 | *F*(1, 35) = 4.32, *p* = 0.045 | Power: y= x^0.10^+ 3.96 |
|  |  |  |  |  |  |  |  |  |  |  |
| Middle Frontal Gyrus | L | -38 | 59 | 12 | 68 | 5.30 | 4.06 |  |  |  |
| Middle Frontal Gyrus | L | -53 | 15 | 41 | 32 | 4.38 | 3.75 |  |  |  |
| Medial Frontal Gyrus | L | -4 | 43 | 28 | 277 | 6.01 | 4.37 |  |  |  |
| Medial Frontal Gyrus | L | 0 | 25 | 40 | 249 | 5.95 | 4.36 |  |  |  |
| Medial Frontal Gyrus | L | -15 | 49 | 14 | 135 | 5.02 | 3.96 |  |  |  |
| Superior Frontal Gyrus | L | -19 | 57 | 27 | 222 | 6.36 | 4.62 |  |  |  |
| Superior Frontal Gyrus | L | 0 | 5 | 56 | 220 | 6.41 | 4.23 | 0.299 | *F*(3, 33) = 4.69, *p* = 0.008 | Cubic: y = -0.61x^3^ + 2.55x^2^ -2.26x + 3.96 |
|  |  |  |  |  |  |  |  | 0.215 | *F*(1, 35) = 9.60, *p* = 0.004 | Linear: y = -0.52x + 3.40 |
|  |  |  |  |  |  |  |  | 0.173 | *F*(1, 35) = 7.33, *p* = 0.010 | Logarithmic: y = 0.43ln(x) + 4.11 |
|  |  |  |  |  |  |  |  | 0.230 | *F*(1, 34) = 5.09, *p* = 0.012 | Quadratic: y = -0.18x^2^ + 1.02x + 3.18 |
|  |  |  |  |  |  |  |  | 0.215 | *F*(1, 35) = 9.57, *p* = 0.004 | Compound: y = 3.29 × 1.15^x^ |
|  |  |  |  |  |  |  |  |  |  | Growth: y = *e*^1.19+0.14x^ |
|  |  |  |  |  |  |  |  |  |  | Exponential: y = *e*^0.14x^ × 3.29 |
| Superior Frontal Gyrus | L | -26 | 43 | 23 | 166 | 5.76 | 4.44 |  |  |  |
| Superior Frontal Gyrus | L | -38 | 53 | 23 | 157 | 6.02 | 4.54 |  |  |  |
| Cingulate Gyrus | L | -7 | -15 | 31 | 214 | 5.91 | 4.20 |  |  |  |
| Cingulate Gyrus | L | -4 | 11 | 32 | 138 | 5.47 | 4.51 |  |  |  |
| Cingulate Gyrus | L | -21 | 13 | 33 | 136 | 4.82 | 3.98 |  |  |  |
| Cingulate Gyrus | L | -19 | -37 | 29 | 123 | 5.31 | 3.97 |  |  |  |
| Cingulate Gyrus | L | -17 | 2 | 40 | 121 | 5.08 | 4.02 |  |  |  |
| Cingulate Gyrus | L | 0 | -1 | 31 | 107 | 4.36 | 3.79 |  |  |  |
| Cingulate Gyrus | L | -19 | -13 | 35 | 66 | 4.15 | 3.72 |  |  |  |
| Insula | L | -31 | 21 | 16 | 145 | 5.52 | 4.33 |  |  |  |
| Insula | L | -34 | -6 | 21 | 81 | 4.50 | 3.76 |  |  |  |
| Insula | L | -42 | -18 | 3 | 27 | 3.80 | 3.51 |  |  |  |
| Insula | L | -39 | -21 | 15 | 28 | 3.56 | 3.42 |  |  |  |
| Claustrum | L | -29 | 15 | -3 | 225 | 7.02 | 4.47 |  |  |  |
| Claustrum | L | -26 | 12 | 13 | 121 | 4.67 | 3.98 |  |  |  |
| Claustrum | L | -34 | -6 | 8 | 83 | 4.67 | 3.77 |  |  |  |
| Precentral Gyrus | L | -49 | -2 | 28 | 121 | 5.30 | 4.13 |  |  |  |
| Precentral Gyrus | L | -43 | -4 | 41 | 111 | 4.94 | 3.95 |  |  |  |
| Precentral Gyrus | L | -37 | 20 | 38 | 108 | 5.34 | 4.09 |  |  |  |
| Precentral Gyrus | L | -32 | -6 | 31 | 75 | 4.81 | 3.85 |  |  |  |
| Precentral Gyrus | L | -42 | -13 | 25 | 72 | 4.53 | 3.79 |  |  |  |
| Precentral Gyrus | L | -42 | 7 | 31 | 67 | 5.12 | 4.15 | 0.208 | *F*(1, 35) = 9.19, *p* = 0.005 | S: y = *e* ^(-0.07/x + 1.46)^ |
|  |  |  |  |  |  |  |  | 0.112 | *F*(1, 35) = 4.41, *p* = 0.043 | Linear: y = 0.40x + 3.45 |
|  |  |  |  |  |  |  |  | 0.135 | *F*(1, 35) = 5.45, *p* = 0.025 | Logarithmic: y = 0.40ln(x) + 3.99 |
|  |  |  |  |  |  |  |  | 0.167 | *F*(1, 35) = 7.01, *p* = 0.012 | Inverse: y = -0.20/x + 4.33 |
|  |  |  |  |  |  |  |  | 0.143 | *F*(1, 35) = 5.86, *p* = 0.021 | Power: y= x^0.13^+3.83 |
| Precentral Gyrus | L | -31 | -17 | 36 | 55 | 4.24 | 3.62 |  |  |  |
| Precentral Gyrus | L | -25 | -16 | 44 | 48 | 3.94 | 3.50 |  |  |  |
| Postcentral Gyrus | L | -33 | -29 | 38 | 59 | 4.03 | 3.55 |  |  |  |
| Postcentral Gyrus | L | -55 | -17 | 33 | 54 | 3.74 | 3.49 |  |  |  |
| Postcentral Gyrus | L | -61 | -14 | 25 | 26 | 4.27 | 3.69 |  |  |  |
| Postcentral Gyrus | L | -50 | -14 | 20 | 16 | 3.48 | 3.40 |  |  |  |
| Inferior Parietal Lobule | L | -50 | -42 | 24 | 198 | 6.48 | 4.37 |  |  |  |
| Inferior Parietal Lobule | L | -58 | -42 | 24 | 146 | 6.49 | 4.39 | 0.159 | *F*(1, 35) = 6.60, *p* = 0.015 | S: y = *e*^(-0.05/x + 1.44)^ |
| Superior Parietal Lobule | L | -27 | -64 | 44 | 56 | 4.62 | 3.82 |  |  |  |
| Superior Parietal Lobule | L | -35 | -54 | 49 | 48 | 4.43 | 3.74 |  |  |  |
| Precuneus | L | -20 | -71 | 19 | 196 | 5.64 | 4.17 |  |  |  |
| Precuneus | L | -16 | -43 | 40 | 23 | 3.89 | 3.49 | 0.212 | *F*(3, 33) = 2.96, *p* = 0.047 | Cubic: y = -0.01x^3^ +0.12x^2^ + 0.34x + 3.26 |
|  |  |  |  |  |  |  |  | 0.210 | *F*(1, 35) = 9.33, *p* = 0.004 | Linear: y = 0.58x + 3.17 |
|  |  |  |  |  |  |  |  | 0.169 | *F*(1, 35) = 7.13, *p* = 0.011 | Logarithmic: y = 0.49ln(x) + 3.96 |
|  |  |  |  |  |  |  |  | 0.212 | *F*(1, 34) = 4.57, *p* = 0.017 | Quadratic: y = 0.06x2 + 0.41x + 3.24 |
|  |  |  |  |  |  |  |  | 0.167 | *F*(1, 35) = 7.03, *p* = 0.012 | Compound: y = 3.05 × 1.17^x^ |
|  |  |  |  |  |  |  |  |  |  | Growth: y = *e*^0.16x + 1.12^ |
|  |  |  |  |  |  |  |  |  |  | Exponential: y = *e*^0.16x^ × 3.05 |
| Precuneus | L | -16 | -60 | 26 | 14 | 3.57 | 3.43 |  |  |  |
| Supramarginal Gyrus | L | -37 | -47 | 32 | 172 | 5.28 | 4.13 |  |  |  |
| Angular Gyrus | L | -31 | -59 | 32 | 144 | 5.29 | 3.97 |  |  |  |
| Middle Temporal Gyrus | L | -45 | -61 | 0 | 214 | 6.21 | 4.78 |  |  |  |
| Middle Temporal Gyrus | L | -32 | -62 | 12 | 126 | 4.93 | 4.10 |  |  |  |
| Middle Temporal Gyrus | L | -40 | -53 | 10 | 120 | 5.73 | 4.45 |  |  |  |
| Middle Temporal Gyrus | L | -48 | -66 | 23 | 114 | 4.77 | 3.73 |  |  |  |
| Superior Temporal Gyrus | L | -53 | -55 | 12 | 148 | 5.34 | 4.24 |  |  |  |
| Superior Temporal Gyrus | L | -56 | -61 | 21 | 54 | 4.46 | 3.84 |  |  |  |
| Superior Temporal Gyrus | L | -40 | -41 | 12 | 50 | 3.76 | 3.53 |  |  |  |
| Fusiform Gyrus | L | -38 | -67 | -9 | 208 | 7.69 | 5.16 |  |  |  |
| Fusiform Gyrus | L | -40 | -52 | -16 | 154 | 5.91 | 4.43 |  |  |  |
| Fusiform Gyrus | L | -40 | -49 | -6 | 145 | 6.20 | 4.64 |  |  |  |
| Fusiform Gyrus | L | -42 | -37 | -12 | 101 | 6.22 | 4.44 |  |  |  |
| Inferior Occipital Gyrus | L | -35 | -78 | -3 | 216 | 7.01 | 4.86 |  |  |  |
| Middle Occipital Gyrus | L | -37 | -73 | 10 | 220 | 6.56 | 4.68 |  |  |  |
| Middle Occipital Gyrus | L | -22 | -86 | 14 | 185 | 5.81 | 4.35 | 0.182 | *F*(1, 34) = 3.78, *p* = 0.033 | Quadratic: y = 0.44x^2^ -0.78x + 3.84 |
|  |  |  |  |  |  |  |  | 0.117 | *F*(1, 35) = 4.62, *p* = 0.039 | Linear: y = 0.46x + 3.28 |
| Cuneus | L | -1 | -78 | 16 | 167 | 5.23 | 4.20 |  |  |  |
| Cuneus | L | -16 | -68 | 10 | 155 | 5.26 | 4.20 | 0.266 | *F*(3, 33) = 3.99, *p* = 0.016 | Cubic: y = -0.01x^3^ +0.12x^2^ + 0.34x + 3.26 |
|  |  |  |  |  |  |  |  | 0.114 | *F*(1, 35) = 4.48, *p* = 0.041 | Linear: y = 0.39x + 3.44 |
|  |  |  |  |  |  |  |  | 0.139 | *F*(1, 35) = 5.67, *p* = 0.023 | Compound: y = 3.25 × 1.13^x^ |
|  |  |  |  |  |  |  |  |  |  | Growth: y = *e*^0.12x + 1.18^ |
|  |  |  |  |  |  |  |  |  |  | Exponential: y = *e*^0.12x^ × 3.25 |
| Cuneus | L | -13 | -92 | 4 | 136 | 5.09 | 4.09 |  |  |  |
| Cuneus | L | -7 | -80 | 22 | 125 | 5.68 | 4.23 |  |  |  |
| Cuneus | L | -10 | -87 | 20 | 94 | 5.17 | 4.20 |  |  |  |
| Cuneus | L | -13 | -81 | 35 | 15 | 3.63 | 3.44 |  |  |  |
| Lingual Gyrus | L | -16 | -83 | -3 | 127 | 5.23 | 4.03 |  |  |  |
| Lingual Gyrus | L | -19 | -55 | 2 | 97 | 4.89 | 3.88 |  |  |  |
| Lentiform Nucleus | L | -21 | -8 | -3 | 251 | 6.36 | 4.30 |  |  |  |
| Caudate | L | -16 | -2 | 13 | 149 | 6.02 | 4.23 |  |  |  |
| Thalamus | L | -21 | -24 | 14 | 178 | 5.50 | 4.04 |  |  |  |
| Thalamus | L | -12 | -10 | 11 | 100 | 4.22 | 3.65 |  |  |  |
| Thalamus | L | -12 | -36 | 18 | 105 | 5.28 | 3.94 |  |  |  |
| Thalamus | L | -3 | -12 | 21 | 82 | 4.63 | 3.75 | 0.171 | *F*(1, 35) = 7.24, *p* = 0.011 | S: y = *e*^(-0.06/x + 1.41)^ |
|  |  |  |  |  |  |  |  | 0.108 | *F*(1, 35) = 4.23, *p* = 0.047 | Logarithmic: y = 0.36ln(x) + 3.88 |
|  |  |  |  |  |  |  |  | 0.153 | *F*(1, 35) = 6.30, *p* = 0.017 | Inverse: y = -0.20/x + 4.21 |
|  |  |  |  |  |  |  |  | 0.109 | *F*(1, 35) = 4.29, *p* = 0.046 | Power: y= x^0.10^ + 3.74 |
| Thalamus | L | -3 | -14 | -1 | 74 | 4.59 | 3.79 |  |  |  |
| Thalamus | L | -18 | -35 | 7 | 56 | 4.44 | 3.74 |  |  |  |
| Thalamus | L | -19 | -13 | 17 | 44 | 3.72 | 3.46 |  |  |  |
| MidbrainRed Nucleus | L | -2 | -22 | -12 | 22 | 3.84 | 3.59 |  |  |  |
| Culmen | L | -17 | -40 | -15 | 28 | 3.80 | 3.51 |  |  |  |
| Culmen | L | -17 | -49 | -18 | 11 | 3.60 | 3.42 |  |  |  |
| Sub-lobar | L | -21 | -21 | -4 | 40 | 4.30 | 3.63 |  |  |  |
| Inferior Frontal Gyrus | R | 51 | 17 | 17 | 244 | 6.58 | 4.62 |  |  |  |
| Inferior Frontal Gyrus | R | 53 | 7 | 31 | 98 | 4.89 | 3.86 |  |  |  |
| Inferior Frontal Gyrus | R | 41 | 31 | 5 | 78 | 4.57 | 3.78 |  |  |  |
| Middle Frontal Gyrus | R | 44 | 37 | 22 | 168 | 6.47 | 4.59 |  |  |  |
| Middle Frontal Gyrus | R | 49 | 27 | 23 | 168 | 5.96 | 4.62 |  |  |  |
| Middle Frontal Gyrus | R | 55 | 35 | 15 | 100 | 5.19 | 4.17 |  |  |  |
| Middle Frontal Gyrus | R | 29 | 34 | 25 | 98 | 4.25 | 3.72 |  |  |  |
| Middle Frontal Gyrus | R | 35 | 41 | 5 | 67 | 5.01 | 3.99 |  |  |  |
| Medial Frontal Gyrus | R | 22 | 43 | 15 | 220 | 5.18 | 3.96 |  |  |  |
| Superior Frontal Gyrus | R | 26 | 51 | 25 | 248 | 6.41 | 4.43 |  |  |  |
| Superior Frontal Gyrus | R | 12 | 59 | 24 | 205 | 5.91 | 4.33 |  |  |  |
| Cingulate Gyrus | R | 4 | 32 | 27 | 280 | 6.33 | 4.76 | 0.128 | *F*(1, 35) = 5.12, *p* = 0.030 | S: y = *e*^(-0.05/x + 1.40)^ |
|  |  |  |  |  |  |  |  | 0.120 | *F*(1, 35) = 4.77, *p* = 0.036 | Logarithmic: y = 0.39ln(x) + 3.88 |
|  |  |  |  |  |  |  |  | 0.124 | *F*(1, 35) = 4.96, *p* = 0.032 | Inverse: y = -0.18/x + 4.17 |
|  |  |  |  |  |  |  |  | 0.122 | *F*(1, 35) = 4.88, *p* = 0.034 | Power: y= x^0.10^ + 3.74 |
| Cingulate Gyrus | R | 21 | 12 | 32 | 216 | 6.96 | 4.44 | 0.138 | *F*(1, 35) = 5.62, *p* = 0.023 | Compound: y = 3.15 × 1.12^x^ |
|  |  |  |  |  |  |  |  |  |  | Growth: y = *e*^0.12x + 1.15^ |
|  |  |  |  |  |  |  |  |  |  | Exponential: y = *e*^0.12x^ × 3.15 |
|  |  |  |  |  |  |  |  | 0.133 | *F*(1, 35) = 5.38, *p* = 0.026 | Linear: y = 0.42x + 3.25 |
|  |  |  |  |  |  |  |  | 0.110 | *F*(1, 35) = 4.31, *p* = 0.045 | Logarithmic: y = 0.35ln(x) + 3.82 |
|  |  |  |  |  |  |  |  | 0.114 | *F*(1, 35) = 4.50, *p* = 0.041 | Power: y= x^0.10^ + 3.69 |
| Cingulate Gyrus | R | 7 | 5 | 40 | 47 | 3.95 | 3.57 |  |  |  |
| Anterior Cingulate | R | 3 | 20 | 24 | 160 | 5.98 | 4.66 |  |  |  |
| Posterior Cingulate | R | 9 | -37 | 20 | 25 | 3.96 | 3.55 | 0.280 | *F*(3, 33) = 4.27, *p* = 0.012 | Cubic: y = -0.66x^3^ + 2.89x^2^ - 2.90x + 3.91 |
|  |  |  |  |  |  |  |  | 0.179 | *F*(1, 35) = 7.64, *p* = 0.009 | Linear: y = 0.43x + 3.17 |
|  |  |  |  |  |  |  |  | 0.148 | *F*(1, 35) = 6.07, *p* = 0.019 | Logarithmic: y = 0.37ln(x) + 3.76 |
|  |  |  |  |  |  |  |  | 0.183 | *F*(1, 34) = 3.80, *p* = 0.032 | Quadratic: y = -0.08x^2^ + 0.65x + 3.07 |
|  |  |  |  |  |  |  |  | 0.157 | *F*(1, 35) = 6.50, *p* = 0.015 | Power: y= x^0.11^ + 3.64 |
|  |  |  |  |  |  |  |  | 0.188 | *F*(1, 35) = 8.12, *p* = 0.007 | Compound: y = 3.03 × 1.14^x^ |
|  |  |  |  |  |  |  |  |  |  | Growth: y = *e*^0.13x + 1.11^ |
|  |  |  |  |  |  |  |  |  |  | Exponential: y = *e*^0.13x^ × 3.03 |
| Insula | R | 40 | -44 | 14 | 105 | 4.71 | 3.77 |  |  |  |
| Insula | R | 31 | -34 | 21 | 58 | 5.42 | 4.20 |  |  |  |
| Insula | R | 51 | -19 | 24 | 49 | 4.41 | 3.61 |  |  |  |
| Insula | R | 28 | -24 | 22 | 35 | 4.66 | 3.73 |  |  |  |
| Claustrum | R | 32 | 1 | 21 | 190 | 5.84 | 4.27 | 0.171 | *F*(1, 34) = 3.80, *p* = 0.032 | Quadratic: y = 0.20x^2^ + 0.11x + 3.28 |
|  |  |  |  |  |  |  |  | 0.166 | *F*(1, 35) = 6.99, *p* = 0.012 | Linear: y = 0.50x + 3.14 |
|  |  |  |  |  |  |  |  | 0.114 | *F*(1, 35) = 4.51, *p* = 0.041 | Logarithmic: y = 0.38ln(x) + 3.83 |
|  |  |  |  |  |  |  |  | 0.144 | *F*(1, 35) = 5.91, *p* = 0.020 | Compound: y = 3.02 × 1.15^x^ |
|  |  |  |  |  |  |  |  |  |  | Growth: y = *e*^0.14x + 1.10^ |
|  |  |  |  |  |  |  |  |  |  | Exponential: y = *e*^0.14x^ × 3.02 |
| Claustrum | R | 35 | 7 | 7 | 185 | 5.46 | 4.26 |  |  |  |
| Claustrum | R | 29 | 8 | 14 | 111 | 5.36 | 4.19 |  |  |  |
| Claustrum | R | 31 | -22 | 10 | 17 | 3.76 | 3.45 |  |  |  |
| Precentral Gyrus | R | 44 | -1 | 26 | 206 | 5.51 | 4.16 | 0.130 | *F*(1, 35) = 5.25, *p* = 0.028 | S: y = *e*^(-0.05/x + 1.38)^ |
| Precentral Gyrus | R | 36 | 7 | 33 | 141 | 5.73 | 4.18 |  |  |  |
| Postcentral Gyrus | R | 28 | -21 | 35 | 51 | 4.76 | 3.76 |  |  |  |
| Inferior Parietal Lobule | R | 42 | -33 | 26 | 39 | 4.73 | 3.90 |  |  |  |
| Inferior Parietal Lobule | R | 50 | -33 | 33 | 21 | 4.40 | 3.72 |  |  |  |
| Precuneus | R | 31 | -73 | 16 | 156 | 5.13 | 4.16 |  |  |  |
| Precuneus | R | 28 | -73 | 30 | 50 | 4.15 | 3.61 |  |  |  |
| Precuneus | R | 22 | -64 | 35 | 27 | 3.83 | 3.51 |  |  |  |
| Angular Gyrus | R | 34 | -58 | 38 | 38 | 4.47 | 3.75 | 0.271 | *F*(3, 33) = 4.10, *p* = 0.014 | Cubic: y = -1.00x^3^ + 4.51x^2^ - 5.18x + 4.73 |
| Middle Temporal Gyrus | R | 45 | -63 | -3 | 281 | 7.20 | 4.90 |  |  |  |
| Middle Temporal Gyrus | R | 40 | -67 | 14 | 221 | 5.77 | 4.24 |  |  |  |
| Middle Temporal Gyrus | R | 31 | -56 | 26 | 95 | 5.19 | 3.95 |  |  |  |
| Superior Temporal Gyrus | R | 48 | -35 | 7 | 48 | 4.36 | 3.69 |  |  |  |
| Superior Temporal Gyrus | R | 49 | 3 | 0 | 46 | 4.18 | 3.67 |  |  |  |
| Superior Temporal Gyrus | R | 53 | -42 | 16 | 45 | 5.15 | 4.01 | 0.280 | *F*(3, 33) = 4.28, *p* = 0.012 | Cubic: y = -0.85x^3^ + 4.02x^2^ - 4.67x + 4.42 |
|  |  |  |  |  |  |  |  | 0.153 | *F*(1, 35) = 6.35, *p* = 0.016 | Linear: y = 0.49x + 3.07 |
|  |  |  |  |  |  |  |  | 0.171 | *F*(1, 34) = 3.50, *p* = 0.042 | Quadratic: y = 0.21x^2^ -0.10x + 3.33 |
|  |  |  |  |  |  |  |  | 0.170 | *F*(1, 35) = 7.17, *p* = 0.011 | Compound: y = 2.86 × 1.17^x^ |
|  |  |  |  |  |  |  |  |  |  | Growth: y = *e*^0.16x + 1.05^ |
|  |  |  |  |  |  |  |  |  |  | Exponential: y = *e*^0.16x^ × 2.86 |
| Temporal Lobe | R | 27 | -42 | -13 | 106 | 4.24 | 3.76 |  |  |  |
| Fusiform Gyrus | R | 37 | -69 | -4 | 280 | 7.21 | 5.03 |  |  |  |
| Middle Occipital Gyrus | R | 27 | -85 | 10 | 162 | 5.12 | 3.94 |  |  |  |
| Cuneus | R | 11 | -88 | 12 | 318 | 5.92 | 4.33 |  |  |  |
| Cuneus | R | 16 | -70 | 16 | 242 | 6.01 | 4.45 |  |  |  |
| Lentiform Nucleus | R | 16 | 3 | 11 | 117 | 5.31 | 3.90 |  |  |  |
| Lentiform Nucleus | R | 19 | -9 | 9 | 117 | 4.67 | 3.85 |  |  |  |
| Lentiform Nucleus | R | 22 | -7 | 0 | 47 | 4.65 | 3.81 |  |  |  |
| Parahippocampal Gyrus | R | 39 | -45 | -5 | 312 | 8.05 | 4.78 |  |  |  |
| Parahippocampal Gyrus | R | 18 | -52 | -1 | 153 | 4.83 | 3.86 |  |  |  |
| Parahippocampal Gyrus | R | 24 | -44 | 7 | 122 | 5.48 | 4.11 |  |  |  |
| Thalamus | R | 21 | -26 | 10 | 78 | 4.70 | 3.71 |  |  |  |
| Thalamus | R | 9 | -18 | 14 | 50 | 4.62 | 3.72 |  |  |  |
| Thalamus | R | 3 | -21 | 8 | 22 | 3.77 | 3.51 |  |  |  |
| Midbrain Red Nucleus | R | 6 | -17 | -10 | 16 | 4.10 | 3.61 |  |  |  |
| Midbrain Subthalamic Nucleus | R | 9 | -14 | -4 | 45 | 5.05 | 3.94 |  |  |  |
| Culmen | R | 21 | -56 | -21 | 158 | 5.31 | 3.92 | 0.229 | *F*(3, 33) = 3.27, *p* = 0.033 | Cubic: y = 0.53x^3^ - 2.36x^2^ + 3.28x + 2.41 |
|  |  |  |  |  |  |  |  | 0.181 | *F*(1, 35) = 7.75, *p* = 0.009 | Linear: y = 0.50x + 3.06 |
|  |  |  |  |  |  |  |  | 0.180 | *F*(1, 35) = 7.66, *p* = 0.009 | Logarithmic: y = 0.46ln(x) + 3.75 |
|  |  |  |  |  |  |  |  | 0.155 | *F*(1, 35) = 6.41, *p* = 0.016 | Inverse: y = -0.20/x + 4.07 |
|  |  |  |  |  |  |  |  | 0.182 | *F*(1, 34) = 3.77, *p* = 0.033 | Quadratic: y = 0.03x^2^ + 0.42x + 3.09 |
|  |  |  |  |  |  |  |  | 0.171 | *F*(1, 35) = 7.21, *p* = 0.011 | Compound: y = 3.02 × 0.13^x^ |
|  |  |  |  |  |  |  |  |  |  | Growth: y = *e*^0.13x + 1.11^ |
|  |  |  |  |  |  |  |  |  |  | Exponential: y = *e*^0.13x^ × 3.02 |
|  |  |  |  |  |  |  |  | 0.184 | *F*(1, 35) = 7.91, *p* = 0.008 | Power: y= x^0.13^ + 3.61 |
|  |  |  |  |  |  |  |  | 0.175 | *F*(1, 35) = 7.41, *p* = 0.010 | S: y = *e*^(-0.06/x + 1.38)^ |
| Culmen | R | 11 | -43 | -12 | 93 | 4.76 | 3.88 |  |  |  |
| Culmen | R | 36 | -51 | -29 | 88 | 5.14 | 3.90 |  |  |  |
| Culmen | R | 32 | -33 | -23 | 65 | 4.85 | 3.75 |  |  |  |
| Declive | R | 31 | -67 | -17 | 137 | 5.07 | 4.02 |  |  |  |
| Tuber | R | 42 | -65 | -27 | 58 | 5.18 | 4.10 |  |  |  |
| Pyramis | R | 28 | -65 | -30 | 75 | 5.51 | 4.21 |  |  |  |
| Sub-Gyral | R | 31 | -47 | 31 | 54 | 4.65 | 3.78 |  |  |  |
| **Cluster2**-Culmen | L | -25 | -30 | -23 | 69 | 4.25 | 3.65 |  |  |  |
| **Cluster3** | L | -53 | -24 | 3 | 32 | 3.94 | 3.51 |  |  |  |
| Superior Temporal Gyrus | L | -53 | -24 | 3 | 22 | 3.94 | 3.53 |  |  |  |
| Superior Temporal Gyrus | L | -53 | -25 | 10 | 10 | 3.85 | 3.47 |  |  |  |

*Note: Only significant models are displayed. For each brain region, the model in the first line shows the best fit if there are several models to be significant.*

**Table 7.** Significant activations for emotional contrasts in the implicit condition and the associations with trait anxiety

| **Significant activations for emotional contrasts in the explicit condition** | | | | | | | | **Associations with trait anxiety** | | |
| --- | --- | --- | --- | --- | --- | --- | --- | --- | --- | --- |
| Regions | Laterality | Talairach coordinate of peak voxel | | | k | *t* value maximum | *t* value average | *R*^2^ | Statistical values | Model(s) |
|  |  | x | y | z |  |  |  |  |  |  |
| **Cluster1** | L/R | -40 | -70 | 2 | 26626 | 9.27 | 4.37 |  |  |  |
| Inferior Frontal Gyrus | L | -34 | 30 | 4 | 238 | 5.31 | 3.96 |  |  |  |
| Inferior Frontal Gyrus | L | -44 | 4 | 29 | 231 | 5.62 | 4.56 |  |  |  |
| Middle Frontal Gyrus | L | -47 | 28 | 18 | 479 | 7.48 | 5.13 |  |  |  |
| Middle Frontal Gyrus | L | -34 | 15 | 26 | 109 | 5.26 | 4.14 |  |  |  |
| Middle Frontal Gyrus | L | -29 | 37 | 24 | 104 | 4.69 | 3.84 |  |  |  |
| Middle Frontal Gyrus | L | -33 | 12 | 48 | 57 | 4.40 | 3.74 |  |  |  |
| Middle Frontal Gyrus | L | -21 | 36 | -4 | 52 | 4.59 | 3.87 |  |  |  |
| Middle Frontal Gyrus | L | -25 | 28 | 28 | 50 | 4.50 | 3.70 |  |  |  |
| Medial Frontal Gyrus | L | -4 | 46 | 25 | 147 | 6.17 | 4.81 |  |  |  |
| Medial Frontal Gyrus | L | -4 | 26 | 38 | 112 | 5.82 | 4.31 | 0.217 | *F*(3, 33) = 3.04, *p* = 0.043 | Cubic: y = -0.75x^3^ + 3.13x^2^ - 3.88x + 5.55 |
|  |  |  |  |  |  |  |  | 0.135 | *F*(1, 35) = 5.48, *p* = 0.025 | Linear: y = -0.54x + 4.90 |
|  |  |  |  |  |  |  |  | 0.106 | *F*(1, 35) = 4.15, *p* = 0.049 | Logarithmic: y = -0.44ln(x) + 4.17 |
| Medial Frontal Gyrus | L | -4 | 38 | 29 | 109 | 5.97 | 4.54 |  |  |  |
| Medial Frontal Gyrus | L | -15 | 46 | 6 | 63 | 4.27 | 3.66 |  |  |  |
| Superior Frontal Gyrus | L | -22 | 39 | 40 | 174 | 5.81 | 4.24 |  |  |  |
| Superior Frontal Gyrus | L | -8 | 13 | 50 | 137 | 4.99 | 4.01 |  |  |  |
| Superior Frontal Gyrus | L | -38 | 47 | 23 | 125 | 5.25 | 4.13 |  |  |  |
| Superior Frontal Gyrus | L | -19 | 54 | 19 | 125 | 4.93 | 4.02 |  |  |  |
| Superior Frontal Gyrus | L | 0 | 54 | 27 | 122 | 5.21 | 4.33 |  |  |  |
| Superior Frontal Gyrus | L | -23 | 51 | 28 | 108 | 5.15 | 4.16 |  |  |  |
| Superior Frontal Gyrus | L | -8 | 28 | 50 | 76 | 4.54 | 3.79 |  |  |  |
| Superior Frontal Gyrus | L | -8 | 36 | 47 | 76 | 4.38 | 3.72 |  |  |  |
| Superior Frontal Gyrus | L | 0 | 64 | 29 | 56 | 5.04 | 3.97 |  |  |  |
| Cingulate Gyrus | L | 0 | 5 | 40 | 168 | 5.07 | 4.17 |  |  |  |
| Cingulate Gyrus | L | -17 | -13 | 43 | 110 | 4.70 | 3.78 |  |  |  |
| Cingulate Gyrus | L | -15 | 26 | 25 | 74 | 6.20 | 4.53 |  |  |  |
| Cingulate Gyrus | L | -7 | -4 | 31 | 73 | 4.39 | 3.67 |  |  |  |
| Cingulate Gyrus | L | -13 | -18 | 32 | 70 | 4.54 | 3.77 |  |  |  |
| Cingulate Gyrus | L | 0 | -26 | 23 | 69 | 4.77 | 3.95 |  |  |  |
| Cingulate Gyrus | L | -18 | 19 | 35 | 25 | 3.94 | 3.65 |  |  |  |
| Anterior Cingulate | L | -4 | 35 | 21 | 166 | 5.89 | 4.61 |  |  |  |
| Anterior Cingulate | L | 0 | 26 | 19 | 113 | 6.23 | 4.48 |  |  |  |
| Posterior Cingulate | L | -3 | -50 | 25 | 217 | 5.50 | 4.08 |  |  |  |
| Posterior Cingulate | L | -12 | -45 | 7 | 132 | 5.51 | 4.29 |  |  |  |
| Posterior Cingulate | L | -3 | -38 | 13 | 128 | 5.18 | 4.09 |  |  |  |
| Posterior Cingulate | L | -9 | -40 | 23 | 72 | 4.91 | 3.93 |  |  |  |
| Insula | L | -35 | 3 | 9 | 160 | 5.61 | 4.17 |  |  |  |
| Insula | L | -34 | -15 | 15 | 142 | 4.67 | 3.84 |  |  |  |
| Insula | L | -33 | 15 | 14 | 135 | 4.59 | 3.84 |  |  |  |
| Insula | L | -45 | -15 | 1 | 113 | 4.83 | 4.00 |  |  |  |
| Insula | L | -37 | -32 | 19 | 48 | 4.18 | 3.60 |  |  |  |
| Claustrum | L | -29 | 12 | -2 | 180 | 6.06 | 4.30 |  |  |  |
| Claustrum | L | -37 | -11 | -4 | 97 | 4.90 | 3.83 |  |  |  |
| Precentral Gyrus | L | -37 | -9 | 48 | 213 | 5.43 | 4.18 |  |  |  |
| Precentral Gyrus | L | -35 | -1 | 31 | 202 | 6.09 | 4.41 |  |  |  |
| Precentral Gyrus | L | -49 | -2 | 14 | 170 | 4.93 | 4.07 |  |  |  |
| Precentral Gyrus | L | -45 | 19 | 36 | 164 | 5.89 | 4.33 |  |  |  |
| Precentral Gyrus | L | -25 | -19 | 41 | 155 | 6.65 | 4.38 |  |  |  |
| Precentral Gyrus | L | -57 | -3 | 21 | 49 | 4.26 | 3.75 |  |  |  |
| Precentral Gyrus | L | -57 | 5 | 5 | 48 | 4.37 | 3.61 |  |  |  |
| Precentral Gyrus | L | -26 | -19 | 60 | 22 | 3.92 | 3.53 |  |  |  |
| Postcentral Gyrus | L | -48 | -14 | 20 | 165 | 5.73 | 4.00 |  |  |  |
| Postcentral Gyrus | L | -44 | -24 | 40 | 83 | 4.63 | 3.82 |  |  |  |
| Inferior Parietal Lobule | L | -33 | -35 | 40 | 250 | 5.83 | 4.14 |  |  |  |
| Inferior Parietal Lobule | L | -44 | -41 | 42 | 200 | 5.37 | 4.07 | 0.264 | *F*(3, 33) = 3.94, *p* = 0.017 | Cubic: y = -0.48x^3^ + 1.53x^2^ -1.15x + 4.22 |
|  |  |  |  |  |  |  |  | 0.231 | *F*(1, 34) = 5.10, *p* = 0.012 | Quadratic: y = -0.65x^2^ + 1.46x + 3.60 |
| Inferior Parietal Lobule | L | -52 | -30 | 34 | 47 | 4.15 | 3.63 |  |  |  |
| Precuneus | L | -22 | -71 | 24 | 349 | 5.51 | 4.39 |  |  |  |
| Precuneus | L | -19 | -60 | 34 | 277 | 6.15 | 4.35 |  |  |  |
| Precuneus | L | -34 | -62 | 36 | 211 | 5.08 | 3.93 |  |  |  |
| Middle Temporal Gyrus | L | -40 | -59 | 21 | 267 | 6.10 | 4.60 |  |  |  |
| Middle Temporal Gyrus | L | -50 | -56 | 10 | 250 | 7.00 | 5.07 |  |  |  |
| Middle Temporal Gyrus | L | -56 | -12 | -6 | 90 | 4.92 | 4.07 |  |  |  |
| Superior Temporal Gyrus | L | -40 | -56 | 12 | 188 | 6.70 | 5.29 |  |  |  |
| Superior Temporal Gyrus | L | -50 | -45 | 21 | 130 | 5.01 | 4.04 |  |  |  |
| Superior Temporal Gyrus | L | -37 | -29 | 7 | 112 | 4.81 | 3.83 |  |  |  |
| Superior Temporal Gyrus | L | -56 | -27 | 3 | 110 | 5.40 | 3.99 |  |  |  |
| Superior Temporal Gyrus | L | -45 | -42 | 14 | 106 | 5.43 | 4.16 |  |  |  |
| Superior Temporal Gyrus | L | -55 | -39 | 8 | 103 | 4.28 | 3.69 |  |  |  |
| Superior Temporal Gyrus | L | -43 | 1 | -12 | 87 | 5.13 | 4.03 | 0.297 | *F*(3, 33) = 4.64, *p* = 0.008 | Cubic: y = -0.33x^3^ + 0.93x^2^ -0.61x + 4.16 |
|  |  |  |  |  |  |  |  | 0.125 | *F*(1, 35) = 5.01, *p* = 0.032 | Linear: y = -0.40x + 4.44 |
|  |  |  |  |  |  |  |  | 0.277 | *F*(1, 34) = 6.50, *p* = 0.004 | Quadratic: y = -0.56x^2^ + 1.18x + 3.74 |
|  |  |  |  |  |  |  |  | 0.143 | *F*(1, 35) = 5.86, *p* = 0.021 | Compound: y = 4.42 × 0.89^x^ |
|  |  |  |  |  |  |  |  |  |  | Growth: y = *e*^0.12x + 1.49^ |
|  |  |  |  |  |  |  |  |  |  | Exponential: y =*e*^0.12x^ × 4.42 |
| Superior Temporal Gyrus | L | -44 | 12 | -12 | 61 | 4.69 | 3.70 |  |  |  |
| Superior Temporal Gyrus | L | -54 | -3 | -6 | 47 | 4.85 | 3.85 |  |  |  |
| Transverse Temporal Gyrus | L | -56 | -19 | 11 | 28 | 3.80 | 3.50 |  |  |  |
| Middle Occipital Gyrus | L | -40 | -70 | 2 | 809 | 9.27 | 6.05 |  |  |  |
| Cuneus | L | -17 | -77 | 12 | 333 | 6.00 | 4.37 |  |  |  |
| Cuneus | L | -16 | -86 | 22 | 137 | 4.53 | 3.82 |  |  |  |
| Lingual Gyrus | L | -13 | -89 | 0 | 226 | 5.28 | 4.01 |  |  |  |
| Lentiform Nucleus | L | -31 | -20 | 5 | 101 | 5.44 | 4.05 |  |  |  |
| Lentiform Nucleus | L | -25 | 3 | -4 | 91 | 5.73 | 4.20 |  |  |  |
| Parahippocampal Gyrus | L | -18 | -35 | 4 | 135 | 5.02 | 3.93 | 0.188 | *F*(1, 34) = 3.94, *p* = 0.029 | Quadratic: y = -0.26x^2^ + 1.14x + 2.97 |
|  |  |  |  |  |  |  |  | 0.154 | *F*(1, 35) = 6.35, *p* = 0.016 | Linear: y = 0.42x + 3.29 |
|  |  |  |  |  |  |  |  | 0.177 | *F*(1, 35) = 7.53, *p* = 0.009 | Logarithmic: y = 0.42ln(x) + 3.87 |
|  |  |  |  |  |  |  |  | 0.127 | *F*(1, 35) = 5.07, *p* = 0.031 | Inverse: y = -0.16/x + 4.13 |
|  |  |  |  |  |  |  |  | 0.155 | *F*(1, 35) = 6.41, *p* = 0.016 | Compound: y = 3.24 × 1.12^x^ |
|  |  |  |  |  |  |  |  |  |  | Growth: y = *e*^0.11x + 1.18^ |
|  |  |  |  |  |  |  |  |  |  | Exponential: y = *e*^0.11x^ × 3.24 |
|  |  |  |  |  |  |  |  | 0.175 | *F*(1, 35) = 7.43, *p* = 0.010 | Power: y= x^0.11^ + 3.76 |
|  |  |  |  |  |  |  |  | 0.123 | *F*(1, 35) = 4.89, *p* = 0.034 | S: y = *e*^(-0.04/x + 1.39)^ |
| Caudate | L | -19 | -5 | 20 | 117 | 5.53 | 4.02 |  |  |  |
| Caudate | L | -13 | 10 | 15 | 38 | 4.38 | 3.72 |  |  |  |
| Thalamus | L | -15 | -16 | 10 | 131 | 4.87 | 3.89 |  |  |  |
| Thalamus | L | -12 | -8 | 9 | 70 | 4.65 | 3.78 |  |  |  |
| Culmen | L | -15 | -50 | -3 | 423 | 5.96 | 4.53 |  |  |  |
| Culmen | L | -6 | -50 | 0 | 189 | 5.92 | 4.41 |  |  |  |
| Culmen of Vermis | L | 0 | -64 | -3 | 129 | 4.53 | 3.73 |  |  |  |
| Uvula | L | -20 | -76 | -23 | 292 | 4.89 | 3.89 |  |  |  |
| Sub-Gyral | L | -42 | -46 | -10 | 802 | 8.24 | 5.27 | 0.292 | *F*(3, 33) = 4.54, *p* = 0.009 | Cubic: y = 0.09x^3^ - 0.89x^2^ + 1.34x + 3.84 |
|  |  |  |  |  |  |  |  | 0.185 | *F*(1, 35) = 7.94, *p* = 0.008 | Linear: y = -0.49x + 4.55 |
|  |  |  |  |  |  |  |  | 0.291 | *F*(1, 34) = 6.97, *p* = 0.003 | Quadratic: y = -0.47x^2^ + 0.84x + 3.96 |
|  |  |  |  |  |  |  |  | 0.182 | *F*(1, 35) = 7.78, *p* = 0.008 | Compound: y = 4.49 × 0.88^x^ |
|  |  |  |  |  |  |  |  |  |  | Growth: y = *e*^-0.13x + 1.50^ |
|  |  |  |  |  |  |  |  |  |  | Exponential: y = *e*^-0.13x^ × 4.49 |
| Sub-Gyral | L | -18 | 4 | 53 | 89 | 4.80 | 3.89 |  |  |  |
| Inferior Frontal Gyrus | R | 56 | 16 | 19 | 269 | 7.26 | 4.81 |  |  |  |
| Inferior Frontal Gyrus | R | 49 | 24 | 21 | 255 | 6.78 | 5.12 |  |  |  |
| Inferior Frontal Gyrus | R | 34 | 26 | 5 | 223 | 5.95 | 4.41 |  |  |  |
| Inferior Frontal Gyrus | R | 41 | 34 | 7 | 147 | 5.39 | 4.19 | 0.179 | *F*(1, 34) = 3.70, *p* = 0.035 | Quadratic: y = -0.28x^2^ + 0.46x + 3.94 |
|  |  |  |  |  |  |  |  | 0.122 | *F*(1, 35) = 4.88, *p* = 0.034 | Linear: y = -0.32x + 4.29 |
|  |  |  |  |  |  |  |  | 0.122 | *F*(1, 35) = 4.87, *p* = 0.034 | Compound: y = 4.24 × 0.92^x^ |
|  |  |  |  |  |  |  |  |  |  | Growth: y = *e*^-0.09x + 1.44^ |
|  |  |  |  |  |  |  |  |  |  | Exponential: y = *e*^-0.09x^ × 4.24 |
| Inferior Frontal Gyrus | R | 36 | 16 | -16 | 76 | 5.27 | 4.04 |  |  |  |
| Middle Frontal Gyrus | R | 32 | 39 | 19 | 153 | 5.30 | 4.06 |  |  |  |
| Middle Frontal Gyrus | R | 32 | 27 | 28 | 144 | 4.85 | 3.96 |  |  |  |
| Middle Frontal Gyrus | R | 26 | -9 | 43 | 31 | 3.81 | 3.49 | 0.262 | *F*(3, 33) = 3.90, *p* = 0.017 | Cubic: y = -0.81x^3^ + 3.22x^2^ - 3.85x + 5.39 |
|  |  |  |  |  |  |  |  | 0.163 | *F*(1, 35) = 6.84, *p* = 0.013 | Linear: y = -0.66x + 4.88 |
|  |  |  |  |  |  |  |  | 0.111 | *F*(1, 35) = 4.36, *p* = 0.044 | Logarithmic: y = -0.50ln(x) + 3.98 |
|  |  |  |  |  |  |  |  | 0.204 | *F*(1, 34) = 4.35, *p* = 0.021 | Quadratic: y = -0.42x^2^ + 0.51x + 4.35 |
|  |  |  |  |  |  |  |  | 0.190 | *F*(1, 35) = 8.23, *p* = 0.007 | Compound: y = 4.90 × 0.81^x^ |
|  |  |  |  |  |  |  |  |  |  | Growth: y = *e*^-0.21x + 1.59^ |
|  |  |  |  |  |  |  |  |  |  | Exponential: y = *e*^-0.21x^ × 4.90 |
|  |  |  |  |  |  |  |  | 0.123 | *F*(1, 35) = 4.92, *p* = 0.033 | Power: y= x^-0.15^ + 3.70 |
| Middle Frontal Gyrus | R | 30 | 0 | 48 | 19 | 3.93 | 3.43 |  |  |  |
| Medial Frontal Gyrus | R | 14 | 13 | 45 | 147 | 5.26 | 4.13 |  |  |  |
| Medial Frontal Gyrus | R | 11 | 1 | 51 | 138 | 4.78 | 3.90 | 0.123 | *F*(1, 35) = 4.90, *p* = 0.033 | Inverse: y = 0.17/x + 3.61 |
| Superior Frontal Gyrus | R | 19 | 54 | 24 | 153 | 5.85 | 4.28 | 0.148 | *F*(1, 35) = 6.06, *p* = 0.016 | Linear: y = -0.44x + 4.49 |
|  |  |  |  |  |  |  |  | 0.123 | *F*(1, 35) = 4.36, *p* = 0.044 | Logarithmic: y = -0.50ln(x) + 3.98 |
|  |  |  |  |  |  |  |  | 0.110 | *F*(1, 35) = 4.33, *p* = 0.045 | Compound: y = 4.32 × 0.90^x^ |
|  |  |  |  |  |  |  |  |  |  | Growth: y = *e*^-0.10x + 1.46^ |
|  |  |  |  |  |  |  |  |  |  | Exponential: y = *e*^-0.10x^ × 4.32 |
| Superior Frontal Gyrus | R | 26 | 51 | 14 | 132 | 5.34 | 4.16 |  |  |  |
| Superior Frontal Gyrus | R | 11 | 42 | 42 | 114 | 5.60 | 4.11 |  |  |  |
| Superior Frontal Gyrus | R | 41 | 40 | 29 | 95 | 4.44 | 3.76 |  |  |  |
| Superior Frontal Gyrus | R | 16 | 48 | 33 | 86 | 4.83 | 3.93 |  |  |  |
| Superior Frontal Gyrus | R | 18 | 27 | 52 | 45 | 4.13 | 3.63 |  |  |  |
| Cingulate Gyrus | R | 7 | 10 | 26 | 113 | 5.50 | 4.16 |  |  |  |
| Cingulate Gyrus | R | 10 | -10 | 31 | 94 | 4.87 | 4.04 |  |  |  |
| Cingulate Gyrus | R | 3 | -21 | 31 | 90 | 5.12 | 4.04 | 0.180 | *F*(1, 34) = 3.73, *p* = 0.034 | Quadratic: y = 0.27x^2^ -0.39x + 3.73 |
|  |  |  |  |  |  |  |  | 0.136 | *F*(1, 35) = 5.49, *p* = 0.025 | Linear: y = 0.37x + 3.39 |
|  |  |  |  |  |  |  |  | 0.108 | *F*(1, 35) = 4.25, *p* = 0.047 | Compound: y = 3.36 × 1.09^x^ |
|  |  |  |  |  |  |  |  |  |  | Growth: y = *e*^0.09x + 1.21^ |
|  |  |  |  |  |  |  |  |  |  | Exponential: y = *e*^0.09x^ × 3.36 |
| Cingulate Gyrus | R | 7 | 20 | 29 | 88 | 5.40 | 4.25 |  |  |  |
| Cingulate Gyrus | R | 19 | -23 | 34 | 53 | 4.38 | 3.67 |  |  |  |
| Cingulate Gyrus | R | 3 | -27 | 39 | 52 | 4.17 | 3.64 |  |  |  |
| Anterior Cingulate | R | 21 | 34 | 8 | 137 | 5.39 | 4.14 |  |  |  |
| Anterior Cingulate | R | 10 | 37 | 1 | 111 | 4.94 | 3.80 |  |  |  |
| Posterior Cingulate | R | 25 | -62 | 21 | 195 | 5.71 | 4.17 | 0.221 | *F*(3, 33) = 3.12, *p* = 0.039 | Cubic: y = -0.23x^3^ + 0.42x^2^ + 0.20x + 3.81 |
|  |  |  |  |  |  |  |  | 0.212 | *F*(1, 34) = 4.58, *p* = 0.017 | Quadratic: y = 1.43x^2^ -0.60x + 3.51 |
| Posterior Cingulate | R | 9 | -55 | 25 | 192 | 5.23 | 4.21 |  |  |  |
| Posterior Cingulate | R | 19 | -54 | 11 | 158 | 4.72 | 3.90 |  |  |  |
| Posterior Cingulate | R | 9 | -34 | 23 | 75 | 4.82 | 3.85 |  |  |  |
| Insula | R | 40 | -23 | 15 | 128 | 4.47 | 3.74 |  |  |  |
| Insula | R | 31 | -34 | 24 | 124 | 5.02 | 3.88 |  |  |  |
| Insula | R | 35 | 1 | 16 | 113 | 5.51 | 3.95 |  |  |  |
| Insula | R | 46 | -10 | 18 | 86 | 4.51 | 3.68 |  |  |  |
| Claustrum | R | 34 | -5 | 2 | 133 | 5.16 | 4.02 |  |  |  |
| Claustrum | R | 29 | 11 | -2 | 99 | 5.59 | 4.22 |  |  |  |
| Claustrum | R | 28 | -7 | 20 | 95 | 5.14 | 4.04 |  |  |  |
| Precentral Gyrus | R | 43 | 15 | 36 | 203 | 5.61 | 4.27 |  |  |  |
| Precentral Gyrus | R | 38 | 3 | 26 | 181 | 5.85 | 4.35 |  |  |  |
| Precentral Gyrus | R | 44 | 0 | 36 | 166 | 4.62 | 4.00 | 0.291 | *F*(3, 33) = 4.51, *p* = 0.009 | Cubic: y = -1.02x^3^ + 4.51x^2^ - 5.70x + 5.57 |
|  |  |  |  |  |  |  |  | 0.124 | *F*(1, 35) = 4.96, *p* = 0.032 | Linear: y = -0.43x + 4.37 |
|  |  |  |  |  |  |  |  | 0.126 | *F*(1, 35) = 5.06, *p* = 0.031 | Logarithmic: y = -0.40ln(x) + 3.77 |
|  |  |  |  |  |  |  |  | 0.110 | *F*(1, 35) = 4.31, *p* = 0.045 | Inverse: y = 0.17/x + 3.49 |
|  |  |  |  |  |  |  |  | 0.122 | *F*(1, 35) = 4.89, *p* = 0.034 | Compound: y = 4.40 × 0.86^x^ |
|  |  |  |  |  |  |  |  |  |  | Growth: y = *e*^-0.15x + 1.48^ |
|  |  |  |  |  |  |  |  |  |  | Exponential: y = *e*^-0.15x^ × 4.40 |
|  |  |  |  |  |  |  |  | 0.105 | *F*(1, 35) = 4.12, *p* = 0.050 | Power: y= x^-0.13^ + 3.58 |
| Precentral Gyrus | R | 46 | -12 | 46 | 144 | 5.91 | 4.21 |  |  |  |
| Precentral Gyrus | R | 55 | -2 | 24 | 109 | 4.50 | 3.80 |  |  |  |
| Precentral Gyrus | R | 38 | -13 | 51 | 104 | 4.91 | 3.93 |  |  |  |
| Precentral Gyrus | R | 58 | -2 | 43 | 38 | 3.90 | 3.58 |  |  |  |
| Precentral Gyrus | R | 31 | -16 | 33 | 23 | 3.56 | 3.42 |  |  |  |
| Precentral Gyrus | R | 26 | -14 | 66 | 19 | 3.75 | 3.52 |  |  |  |
| Precentral Gyrus | R | 61 | 0 | 33 | 18 | 3.52 | 3.40 |  |  |  |
| Postcentral Gyrus | R | 65 | -20 | 24 | 136 | 6.16 | 4.23 |  |  |  |
| Postcentral Gyrus | R | 53 | -25 | 31 | 117 | 4.69 | 3.86 |  |  |  |
| Postcentral Gyrus | R | 56 | -27 | 41 | 80 | 4.97 | 3.93 |  |  |  |
| Postcentral Gyrus | R | 31 | -25 | 32 | 46 | 4.77 | 3.82 |  |  |  |
| Paracentral Lobule | R | 10 | -37 | 47 | 52 | 4.18 | 3.60 |  |  |  |
| Inferior Parietal Lobule | R | 44 | -31 | 49 | 100 | 4.79 | 3.78 |  |  |  |
| Inferior Parietal Lobule | R | 45 | -35 | 36 | 72 | 4.76 | 3.79 |  |  |  |
| Superior Parietal Lobule | R | 34 | -51 | 48 | 12 | 3.68 | 3.51 |  |  |  |
| Precuneus | R | 16 | -68 | 28 | 182 | 5.28 | 4.08 |  |  |  |
| Precuneus | R | 28 | -70 | 37 | 89 | 4.49 | 3.78 |  |  |  |
| Precuneus | R | 19 | -42 | 31 | 58 | 4.28 | 3.68 |  |  |  |
| Supramarginal Gyrus | R | 61 | -48 | 27 | 26 | 4.23 | 3.66 |  |  |  |
| Middle Temporal Gyrus | R | 40 | -62 | 14 | 321 | 6.39 | 4.91 |  |  |  |
| Middle Temporal Gyrus | R | 61 | -52 | -2 | 111 | 5.04 | 4.08 | 0.138 | *F*(1, 35) = 5.62, *p* = 0.023 | Linear: y = -0.38x + 4.31 |
|  |  |  |  |  |  |  |  | 0.107 | *F*(1, 35) = 4.19, *p* = 0.048 | Logarithmic: y = -0.31ln(x) + 3.80 |
|  |  |  |  |  |  |  |  | 0.121 | *F*(1, 35) = 4.82, *p* = 0.035 | Compound: y = 4.22 × 0.91^x^ |
|  |  |  |  |  |  |  |  |  |  | Growth: y = *e*^-0.10x + 1.44^ |
|  |  |  |  |  |  |  |  |  |  | Exponential: y1 = *e*^-0.10x^ × 4.22 |
| Superior Temporal Gyrus | R | 48 | -38 | 7 | 264 | 6.33 | 4.77 |  |  |  |
| Superior Temporal Gyrus | R | 48 | -17 | -6 | 223 | 5.94 | 4.28 |  |  |  |
| Superior Temporal Gyrus | R | 53 | -30 | 8 | 161 | 5.73 | 4.15 | 0.145 | *F*(1, 35) = 5.94, *p* = 0.020 | Linear: y = -0.47x + 4.50 |
|  |  |  |  |  |  |  |  | 0.125 | *F*(1, 35) = 5.01, *p* = 0.032 | Logarithmic: y = -0.41ln(x) + 3.85 |
|  |  |  |  |  |  |  |  | 0.122 | *F*(1, 35) = 4.88, *p* = 0.034 | Compound: y = 4.34 × 0.89^x^ |
|  |  |  |  |  |  |  |  |  |  | Growth: y = *e*^-0.12x + 1.47^ |
|  |  |  |  |  |  |  |  |  |  | Exponential: y = *e*^-0.12x^ × 4.34 |
| Superior Temporal Gyrus | R | 48 | -56 | 25 | 136 | 4.69 | 4.02 |  |  |  |
| Superior Temporal Gyrus | R | 45 | 13 | -15 | 124 | 6.04 | 4.51 |  |  |  |
| Superior Temporal Gyrus | R | 52 | 1 | -5 | 86 | 4.34 | 3.78 |  |  |  |
| Superior Temporal Gyrus | R | 48 | -42 | 23 | 81 | 4.23 | 3.65 |  |  |  |
| Superior Temporal Gyrus | R | 49 | 3 | 5 | 33 | 4.13 | 3.52 |  |  |  |
| Superior Temporal Gyrus | R | 61 | -39 | 22 | 31 | 3.81 | 3.48 |  |  |  |
| Fusiform Gyrus | R | 39 | -53 | -11 | 306 | 9.20 | 6.60 |  |  |  |
| Fusiform Gyrus | R | 39 | -44 | -13 | 280 | 8.81 | 5.61 |  |  |  |
| Middle Occipital Gyrus | R | 40 | -74 | 2 | 281 | 7.73 | 5.35 |  |  |  |
| Middle Occipital Gyrus | R | 48 | -69 | 9 | 264 | 7.76 | 5.48 |  |  |  |
| Cuneus | R | 6 | -73 | 18 | 350 | 5.88 | 4.35 |  |  |  |
| Cuneus | R | 12 | -85 | 8 | 272 | 6.09 | 4.69 |  |  |  |
| Cuneus | R | 15 | -88 | 23 | 138 | 4.93 | 3.92 |  |  |  |
| Cuneus | R | 24 | -80 | 31 | 136 | 5.47 | 4.24 |  |  |  |
| Lingual Gyrus | R | 21 | -83 | 6 | 222 | 5.63 | 4.24 |  |  |  |
| Lingual Gyrus | R | 22 | -76 | -6 | 207 | 4.80 | 3.98 | 0.261 | *F*(3, 33) = 3.89, *p* = 0.017 | Cubic: y = -0.56x^3^ + 2.10x^2^ - 2.42x + 4.70 |
|  |  |  |  |  |  |  |  | 0.172 | *F*(1, 35) = 7.27, *p* = 0.011 | Linear: y = -0.62x + 4.53 |
|  |  |  |  |  |  |  |  | 0.105 | *F*(1, 35) = 4.12, *p* = 0.050 | Logarithmic: y = -0.45ln(x) + 3.67 |
|  |  |  |  |  |  |  |  | 0.227 | *F*(1, 34) = 4.99, *p* = 0.013 | Quadratic: y = -0.44x^2^ + 0.64x + 3.97 |
|  |  |  |  |  |  |  |  | 0.207 | *F*(1, 35) = 9.12, *p* = 0.005 | Compound: y = 4.68 × 0.79^x^ |
|  |  |  |  |  |  |  |  |  |  | Growth: y = *e*^-0.23x + 1.54^ |
|  |  |  |  |  |  |  |  |  |  | Exponential: y = *e*^-0.23x^ × 4.68 |
| Lentiform Nucleus | R | 19 | -1 | 6 | 92 | 5.17 | 4.09 |  |  |  |
| Parahippocampal Gyrus | R | 25 | -56 | -1 | 246 | 5.82 | 4.49 |  |  |  |
| Parahippocampal Gyrus | R | 15 | -39 | 4 | 167 | 5.38 | 4.29 | 0.258 | *F*(3, 33) = 3.83, *p* = 0.018 | Cubic: y = -0.95x^3^ + 4.29x^2^ - 5.36x + 5.23 |
| Parahippocampal Gyrus | R | 33 | -18 | -11 | 91 | 4.52 | 3.78 | 0.107 | *F*(1, 35) = 4.19, *p* = 0.048 | Compound: y = 4.19 × 0.90^x^ |
|  |  |  |  |  |  |  |  |  |  | Growth: y = *e*^-0.11x + 1.43^ |
|  |  |  |  |  |  |  |  |  |  | Exponential: y = *e*^-0.11x^ × 4.19 |
| Parahippocampal Gyrus | R | 23 | -25 | -10 | 53 | 4.40 | 3.84 | 0.163 | *F*(1, 34) = 3.32, *p* = 0.048 | Quadratic: y = -0.24x^2^ + 0.08x + 4.16 |
|  |  |  |  |  |  |  |  | 0.149 | *F*(1, 35) = 6.11, *p* = 0.018 | Linear: y = -0.59x + 4.46 |
|  |  |  |  |  |  |  |  | 0.120 | *F*(1, 35) = 4.77, *p* = 0.036 | Logarithmic: y = -0.49ln(x) + 3.65 |
|  |  |  |  |  |  |  |  | 0.136 | *F*(1, 35) = 5.51, *p* = 0.025 | Compound: y = 4.39 × 0.82^x^ |
|  |  |  |  |  |  |  |  |  |  | Growth: y = *e*^-0.20x + 1.48^ |
|  |  |  |  |  |  |  |  |  |  | Exponential: y = *e*^-0.20x^ × 4.39 |
| Caudate | R | 16 | 0 | 19 | 61 | 3.98 | 3.57 |  |  |  |
| Thalamus | R | 22 | -17 | 15 | 170 | 6.10 | 4.50 |  |  |  |
| Thalamus | R | 24 | -26 | 7 | 151 | 4.93 | 3.95 |  |  |  |
| Thalamus | R | 9 | -9 | 3 | 42 | 4.38 | 3.68 | 0.221 | *F*(3, 33) = 3.11, *p* = 0.039 | Cubic: y = -0.31x^3^ + 0.73x^2^ + 0.05x + 3.50 |
|  |  |  |  |  |  |  |  | 0.205 | *F*(1, 34) = 4.39, *p* = 0.020 | Quadratic: y = -0.67x^2^ + 1.72x + 3.11 |
| Culmen | R | 24 | -57 | -19 | 279 | 6.39 | 4.82 |  |  |  |
| Culmen | R | 11 | -41 | -7 | 182 | 5.01 | 3.95 |  |  |  |
| Culmen | R | 42 | -43 | -28 | 100 | 5.20 | 3.99 |  |  |  |
| Cerebellar Tonsil | R | 34 | -57 | -32 | 51 | 4.03 | 3.76 |  |  |  |
| Declive | R | 35 | -67 | -22 | 228 | 5.79 | 4.49 |  |  |  |
| Pyramis | R | 12 | -69 | -23 | 347 | 7.37 | 4.48 |  |  |  |
| Sub-Gyral | R | 48 | -46 | -5 | 204 | 8.41 | 5.78 |  |  |  |
| Sub-Gyral | R | 31 | -43 | 34 | 103 | 4.72 | 3.89 |  |  |  |
| Sub-Gyral | R | 31 | -58 | 38 | 71 | 4.47 | 3.72 |  |  |  |

*Note: Only significant models are displayed. For each brain region, the model in the first line shows the best fit if there are several models to be significant.*

**Table 8.** Significant activations for the interaction between emotion and task

| Regions | Laterality | Talairach coordinates of peak voxel | | | | k | *t* value maximum | *t* value average |
| --- | --- | --- | --- | --- | --- | --- | --- | --- |
|  |  | x | y | | z |  |  |  |
| ***(Explicit-threat - explicit-neutral) > (implicit-threat - implicit-neutral)*** | | | | | | | | |
| No brain regions were activated. |  |  |  |  | |  |  |  |
| ***(Implicit-threat - implicit-neutral) > (explicit-threat - explicit-neutral)*** | | | | | | | | |
| **Cluster1** | R | 42 | -34 | 52 | | 174 | -5.26 | -3.65 |
| Precentral Gyrus | R | 40 | -16 | 56 | | 27 | -3.53 | -3.25 |
| Precentral Gyrus | R | 40 | -16 | 56 | | 27 | -3.53 | -3.25 |
| Precentral Gyrus | R | 32 | -15 | 62 | | 20 | -3.46 | -3.22 |
| Postcentral Gyrus | R | 42 | -34 | 52 | | 102 | -5.26 | -3.91 |
| Postcentral Gyrus | R | 56 | -27 | 41 | | 25 | -3.64 | -3.34 |
| **Cluster2**-Uvula | L | -13 | -78 | -23 | | 80 | -4.36 | -3.57 |
| **Cluster3**-Middle Temporal Gyrus | R | 63 | -52 | 1 | | 47 | -4.25 | -3.50 |
| Middle Temporal Gyrus | R | 63 | -52 | 1 | | 33 | -4.25 | -3.58 |
| Sub-Gyral | R | 50 | -46 | -5 | | 14 | -4.08 | -3.31 |
| **Cluster4**-Superior Temporal Gyrus | R | 45 | 16 | -18 | | 23 | -4.07 | -3.41 |
| **Cluster5**-Cingulate Gyrus | R | 9 | -52 | 28 | | 23 | -3.97 | -3.41 |
| Cingulate Gyrus | R | 9 | -52 | 28 | | 16 | -3.97 | -3.46 |
| **Cluster6**-Cingulate Gyrus | R | 7 | -41 | 44 | | 20 | -3.90 | -3.43 |
| **Cluster7**-Fusiform Gyrus | R | 42 | -47 | -13 | | 24 | -3.85 | -3.36 |
| Fusiform Gyrus | R | 42 | -47 | -13 | | 16 | -3.85 | -3.38 |
| **Cluster8**-Superior Temporal Gyrus | R | 51 | -56 | 16 | | 22 | -3.80 | -3.38 |
| **Cluster9**-Parahippocampal Gyrus | L | -27 | -36 | -8 | | 15 | -3.71 | -3.35 |
| **Cluster10**-Middle Temporal Gyrus | L | -53 | -11 | -11 | | 22 | -3.62 | -3.30 |
| **Cluster11**-Middle Temporal Gyrus | R | 57 | -6 | -5 | | 11 | -3.43 | -3.27 |
| **Cluster12**-Middle Temporal Gyrus | L | -53 | -11 | -11 | | 22 | -3.62 | -3.30 |
| **Cluster16**-Middle Temporal Gyrus | R | 57 | -6 | -5 | | 11 | -3.43 | -3.27 |


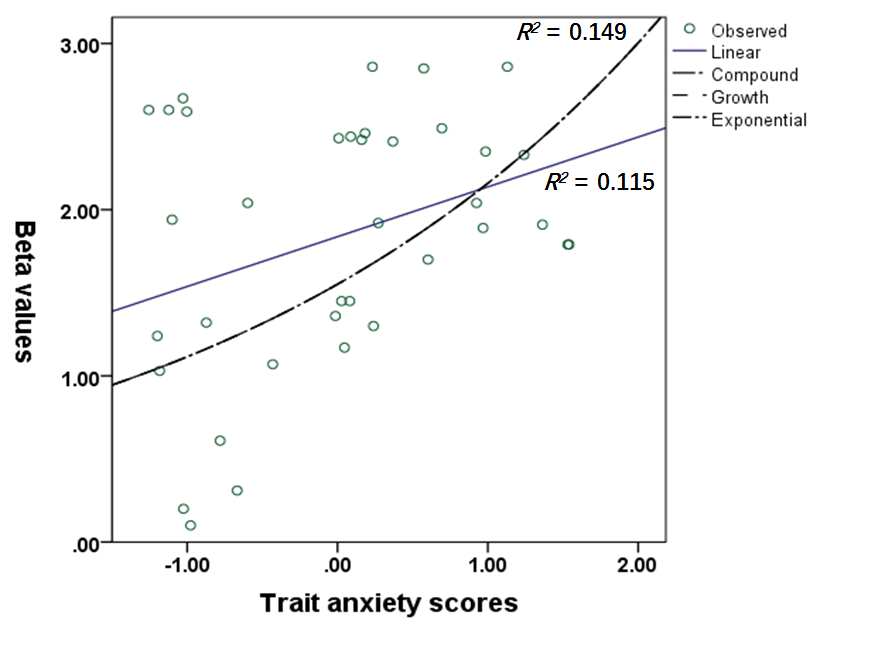


**Figure 3.** Other relations (i.e., linear, compound, growth and exponential) between trait anxiety and right amygdalar responses to threat vs. neutral pictures in the explicit condition, except the cubic relation. Note that the compound, growth and exponential relations do not differ with each other.
